# Study on the structural function and motion performance of pneumatic flexible tree-climbing robot

**DOI:** 10.1371/journal.pone.0323335

**Published:** 2025-06-11

**Authors:** Hongbo Liu, Zhipan Gou

**Affiliations:** 1 Engineering Training Center, Beihua University, Jilin, Jilin, China; 2 School of Mechanical Engineering, Beihua University, Jilin, Jilin, China; King Fahd University of Petroleum & Minerals, SAUDI ARABIA

## Abstract

To enhance the adaptability of tree-climbing robots to changes in tree diameter and load capacity, an “I-shaped” pneumatic flexible tree - climbing robot was designed using self-developed pneumatic flexible joints and retractable needle anchors. The robot consists of an upper waist, a lower waist, a front leg, a rear leg, a load plate, and anchors. Through pneumatic driving, it can achieve efficient and stable driving during tree - climbing and stable grasping trees of varying diameters. Static mechanical models of waist and leg joints were established, the deformation characteristics of joints under different pressures were obtained, and relevant experimental verification was carried out. The kinematic analysis of the robot was performed using a 3D motion capture system, and the motion performance of the robot was obtained under different air pressures, tree diameters, tree inclination angles, and load conditions. The experimental results show that, according to the gait planning and its control sequence for climbing trees, the robot can carry loads to complete functions of climbing upwards, climbing downwards, and crossing obstacles at different inclination angles by alternately coordinating waist deformation and leg clamping through the air pressure control system. The robot can adapt to climbing different types of trees with diameters ranging from 146.4 mm to 292.8 mm, and its maximum load capacity is 2.0 kg. When encircling a tree with a diameter of 290 mm, the maximum horizontal climbing speed in the no-load state is 43.8 mm/min, and the maximum upward climbing speed when climbing vertically is 27 mm/min, while the maximum downward climbing speed is 63 mm/min.

## 1 Introduction

As the complexity and frequency of human-robot interaction continue to increase, robots are expected to possess higher compliance, adaptability and safety [1]. Traditional rigid robots can indeed achieve complex movements through a large number of rigid joints, and use sensing and control technology to make the output force variable and controllable, or wrap rigid components with flexible material skin to improve the flexibility of the robot. However, such robots typically have complex structures, poor adaptability, low safety, and high production costs. There are numerous challenges in applications such as grasping complex and fragile objects, non- structural environment operations, and rehabilitation medicine [2]. Compared with traditional rigid robots, flexible robots are mainly composed of flexible elastic materials, and have good environmental adaptability, and body deformability characteristics, which compensate for the shortcomings of traditional rigid robots. Inspired by the structural characteristics and motion mechanisms of organisms in nature, domestic and foreign scholars have developed a variety of flexible robots [3–6]. These robots can perform functions that are difficult for traditional rigid robots to accomplish and have become a hot spot and development frontier of robot research.

As an important branch of special robots, tree -climbing robots can replace people to do dangerous work in high-altitude complex environments, and complete tasks such as tree monitoring, fruit picking, tree pruning, and cleaning of urban pole-shaped buildings. Their application prospects have attracted extensive attention from scholars around the world, and a large number of tree - climbing robots have emerged [7]. Carnegie Mellon University in the United States developed a biomimetic multi-joint snake-like robot driven by electric motors, with a fuselage diameter of approximately 0.051 m, a total length of 0.94 m, and a weight of 2.9 kg. The robot adopts a modular design, which can flexibly increase or decrease the number of modules according to actual needs, and can be powered by servo motors according to pre-set gait planning, but cannot climb trees with thicker diameters [8]. Shanghai Jiao Tong University developed a snake-shaped robot and innovatively proposed a P-R module with the function of a universal joint, which can make the snake robot more flexibly attached to the external wall of the climbing object, and can adjust the gait and posture more arbitrarily, and can be closely attached to the wrapped object [9]. The above two types of snake-shaped bionic robots can adapt well to changes in tree diameter, but their rotating and ascending climbing methods result in slower movement speeds compared to other biomimetic robots. Moreover, their modular joints will lead to certain difficulties in maintenance. The Shenyang Institute of Automation, Chinese Academy of Sciences designed a new type of climbing robot [10]. On the premise of minimizing the driving source, it can achieve stable multi-fingered grasping and wheeled movement through two motors, and has the ability to stably climb pole-shaped objects of different shapes. Jiangnan University designed a multi-posture and variable-diameter tree-climbing robot with an overall size of 0.4 m × 0.4 m × 0.4 m and a self-weight of about 20 kg [11]. It relies on the friction of rubber wheels to realize actions such as moving straight, rotating and reversing, with a minimum linear motion speed of 0.2 m/s and a rotational angular velocity of up to 1.2 rad/s. Universiti Sains Malaysia developed a pole climbing robot, which consists of a spinal structure with four-bar linkage, and there are two grippers at both ends of the spine [12]. The robot can achieve a motion similar to that of an inchworm according to a pre-programmed gait, with an average speed of 4.87 mm/s. But there is no structure designed to store batteries, and the climbing distance is affected by the battery life. The Hefei Research Institute of the Chinese Academy of Sciences designed a quadruped tree-climbing robot [13]. The foot end of the robot adopts bionic hooks arranged with 3D printing dense array and bionic bonding materials prepared by polymer printing lithography technology for grasping. There are four axial adjustment servos at the arm ends respectively, which can make the robot cling tightly to the tree trunk and maintain a sufficient contact area. Inspired by the movement of octopus, Hosei University developed a pneumatic soft climbing robot, which consists of a three-dimensional curved torso and eight unidirectionally curved legs, weighing 0.55 kg [14]. By controlling the combination of air pressures in the trunk and legs, the robot can climb on various columnar objects. However, the body of this robot is made entirely of silicone, and there is a risk of rupture when the air pressure is too high or when it encounters sharp objects. Based on the flexibility characteristics of modular pneumatic artificial muscles, Leng et al [15] of Harbin Institute of Technology developed a climbing robot using a simple mesh curling and splicing stacking method. By changing the control strategy, more motion modes can be achieved, the maximum load of the vertical climbing tube is 3.0 kg, and the angular speed of the rotating climbing tube is 1.6°/s. However, the robot adopts a completely closed structure and has poor obstacle-crossing ability. Zang et al [16] designed a snake-like winding pneumatic climbing robot, which is mainly composed of two winding soft actuators and one telescopic soft actuator. This robot can achieve efficient climbing movements on rods with different friction surfaces, rods with nearly right angles and rods with variable diameters. Its maximum vertical climbing speed is 30.85 mm/s and its effective load is 500 g. At the same year, this team developed a pneumatic quadruped pole climbing robot, which consists of four bending actuators and one telescopic actuator [17]. By controlling the alternating deformations of the two types of actuators, the pole-climbing movement can be achieved. When climbing the 90° pole, the maximum speed is 2.33 mm/s, and when climbing the horizontal pole at 9 mm/s, the maximum payload is 3.7 times its own weight. The Italian Institute of Technology developed a soft growth robot with adaptive behaviors, which mimics the top buds of climbing plants and senses and coordinates additive adaptive growth through embedded additive manufacturing mechanisms and sensing tips. When encountering dense forests or cluttered areas, robots can adjust their material printing parameters and navigate around obstacles like vines [18]. Xie et al. [19] designed a millimeter-scale continuum robot, which is composed of two variable stiffness phase change core components. Under the precise guidance of a programmable external magnetic field, the robot performs periodic, tip-based elongation in alternating phase change to adapt to climbing and curled branches in a complex environment. Tampere University developed a wireless light-driven climbing robot based on liquid crystal elastomers [20]. By means of the alternating deformations of the grippers at both ends and the body driven by light, the robot can climb on the surfaces of glass, wood, metal, plastic and tubular objects with different cross-sections, and its maximum load capacity is three times its own weight. Guangdong University of Technology proposed a hook and claw soft end gripper driven by shape memory alloy (SMA) [21], which can generate a clamping force of 9.1 N, equivalent to 10 times its own gravity. It can be stably attached to different object surfaces and can ensure the operation of the climbing robot under different postures.

In summary, the driving methods of tree-climbing robots are mainly motor-driven, pneumatic-driven, intelligent material-driven and magnetic field-driven, etc. Among them, the tree-climbing robots driven by motors have a relatively large mass, consume energy quickly, have limited overload capacity, and are mostly composed of rigid joints, which restricts their flexibility. The tree-climbing robots driven by functional materials have such drawbacks as limited driving capacity, poor environmental adaptability, high cost, and low energy conversion efficiency, which restricts their application and development. Pneumatic drive has the characteristics of light weight, high efficiency, no pollution, and strong environmental adaptability, so it is widely used in the design of tree-climbing robots [22]. The grasping operations and attachment methods of tree-climbing robots mainly include the encircling type, hook and claw type, clamping type and winding type. The encircling type offers good stability but has poor ability to cross safety obstacles and is suitable for trees without branches. The hook and claw type features a fast climbing speed and a strong obstacle-crossing ability. However, it will leave irreparable climbing marks on the surfaces of trees. The clamping type is fast in climbing and highly flexible, yet its control system is complex. The winding type can adapt to trees with different friction surfaces, shapes and varying diameters. Nevertheless, its upward rotating motion mode results in a relatively slow crawling speed and the modular joints increase the maintenance difficulty. Each of the above-mentioned attachment methods has its own advantages and disadvantages. In actual structural design, rather than adopting a single constraint attachment form, the advantages of different attachment modes are integrated into the design of tree-climbing robots as needed. This is done to achieve the goal of complementary advantages and performance balance.

Nevertheless, the currently developed pneumatic flexible robots have the following two major deficiencies: (1) insufficient body driving capabilities; (2) inadequate end attachment capabilities. These deficiencies lead to the problems that the pneumatic flexible tree-climbing robots have a low climbing speed, weak load-bearing capacity, and weak ability to resist changes in external loads. To address the problem of unexpected passive deformation of the robot’s body, which is induced by gravity or external force loads during the crawling process, an enhanced pneumatic spatial bending flexible joint is adopted as the waist. This effectively meets the requirement for efficient and stable actuation in climbing motion. Simultaneously, by implementing a series - connected structure for the upper and lower waists, the robot’s flexibility is significantly enhanced. To enhance the robot’s efficient and stable attachment to the rough tree surface during the climbing process and improve its resilience to external load fluctuations, a rigid - flexible coupling leg structure is developed. This structure is designed by a dual-muscle-driven pneumatic unidirectional bending joint in series with a retractable needle anchor. Through air pressure control, the leg structure can adapt to changes in the tree diameter and can adjust the anchoring force in real time in response to load variations, and achieve stable grasping through multiple soft and hard point contacts.

Through the research on the two core issues of the body driving and end attachment of the climbing robot, an “I-shaped” pneumatic flexible tree-climbing robot is developed based on the independently developed waist joint and leg structure. The robot’s structure and function are elaborated in detail, and the deformation, driving force, and anchoring force of the waist joint and leg under different air pressures are experimentally tested. The gaits of the robot during tree climbing and obstacle-crossing are planned, and the motion performance of the robot is tested by a 3D motion capture system.

## 2 Structure and function of pneumatic flexible tree-climbing robot

**Fig 1:**
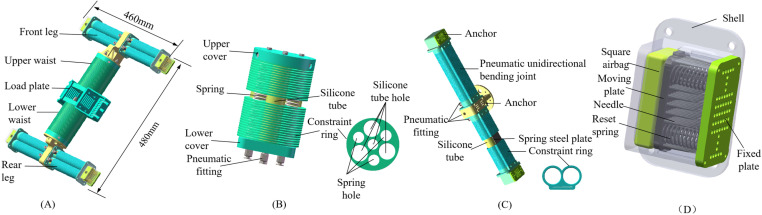
Three-dimensional model. (A) Tree-climbing robot. (B) The enhanced pneumatic flexible spatial bending joint. (C) The dual-muscle-driven pneumatic unidirectional bending joint. (D) Anchor.

The pneumatic flexible tree-climbing robot primarily consists of an upper waist, a lower waist, a front leg, a rear leg, a load plate and connecting parts. Its overall length is 480 mm, width is 460 mm, and total mass is 2.8 kg, as depicted in Fig 1A. The waist is directly driven by the enhanced pneumatic flexible spatial bending joint, which has three degrees of freedom. The upper and lower waist are connected in series via the load plate, which enables the robot to achieve axial elongation and spatial bending. The legs are composed of two grippers that are symmetrically distributed with respect to a plane and an abdominal pneumatic retractable needle anchor, with a total of two degrees of freedom, as shown in Fig 1C. Among them, each gripper is composed of a dual muscle driven pneumatic flexible unidirectional bending joint [23] and an end pneumatic retractable needle anchor connected in series. The developed robot adopts an external control system, with a total of 10 degrees of freedom and a load capacity of 2.0 kg. Through the air pressure control system, the alternating driving of the waist deformation and leg clamping is coordinated to realize the robot’s functions of climbing trees in a straight line and crossing obstacles.

**Fig 2:**
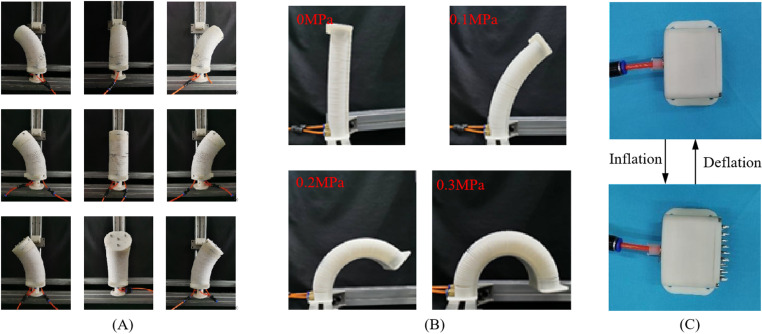
Deformation posture of joints and anchor. (A) The enhanced pneumatic flexible spatial bending joint. (B) The dual-muscle-driven pneumatic unidirectional bending joint. (C) Anchor.

The enhanced pneumatic flexible spatial bending joint consists of three symmetrical and evenly distributed pneumatic artificial muscles and elastic skeletons that form an angle of 120 ° between each other, along with a central enhanced artificial muscle, as shown in Fig 1B. The artificial muscle is a closed cavity formed between the airbag and the end cap, with a set of thin sheet-shaped constraint rings on the outer side. An elastic skeleton is added between the muscles, and it plays a role in supporting and connecting, improving the stiffness and elastic recovery of the joint and ensuring the realization of the joint movement function. When compressed air is injected, the inner wall of the airbag of the artificial muscle is pressurized and expands. Due to the radial restraint of the constraint ring on the outer side, an axial force is generated to drive the joint to move. As depicted in Fig 2A, when the same air pressure is simultaneously injected into the three muscles on the outer ring of the joint, the joint elongates axially. When different air pressure combinations are injected into the three muscles, the joint elongates while bending in different spatial directions. The artificial muscle located at the center of the joint mainly functions to enhance deformation.

The dual-muscle-driven pneumatic unidirectional bending joint produces an approximate circular arc bending under the action of air pressure, which can closely adhere to the surface of the tree and ensure sufficient contact area, as depicted in Fig 2B. After pressure relief, the joint is reset and detached from the tree surface under the dual action of silicone tube and spring steel plate. Therefore, the leg deformation can be adjusted by controlling the air pressure to adapt to the embrace and clamping of trees with different diameters.

To further enhance the anchoring between the leg and the tree surface, pneumatic anchors are added to the abdomen of the leg and the end of the gripper. The anchor consists of a shell, a square airbag, a moving plate, a densely arranged needle punched array, reset springs, and a fixed plat, as depicted in Fig 1D. The square airbag is formed by mixing and pouring “Red Leaf” liquid silicone in a 1:1 ratio. The airbag and telescopic device are assembled inside the shell, and the shell is sealed with the fixed plate by bolts. The air bag is installed at the bottom of the shell and connected to the air pipe through a through-hole at the bottom of the shell. The moving plate is in contact with the upper end face of the airbag. Needle holes and spring holes are designed on the moving plate, and the needles and springs are connected with the moving plate as a whole by interference fit. Similarly, matching blind spring holes and needle through-holes are also set on the fixed plate.

As depicted in Fig 2C, when compressed gas is injected into the airbag of the anchor, the inner wall of the airbag expands under pressure. Meanwhile, the deformation in the bottom and surrounding directions is restricted by the shell. The airbag can only expand towards the top surface, thus pushing the moving plate to move. As the air pressure rises, the needles on the moving plate protrude through the through-holes of the fixed plate and make contact with the surface of the tree, fulfilling the purpose of anchoring. Generally speaking, the more air pressure injected into the square airbag, the more robust the anchoring force becomes. Once the pressure is released, under the combined action of the airbag and the reset spring, the needles will detach from the tree surface and revert to its initial position.

## 3 Theoretical modeling of joints and anchor

### 3.1 Force and deformation analysis of the enhanced pneumatic flexible spatial bending joint

After different compressed gases are injected, the upper cover of the joint is simultaneously affected by the combined action of axial force and bending moment, resulting in composite deformation. The axial force determines the elongation of the joint, and the resultant moment determines the angle size and direction of the joint’s bending.

#### 3.1.1 Axial force and deformation of the joint.

**Fig 3:**
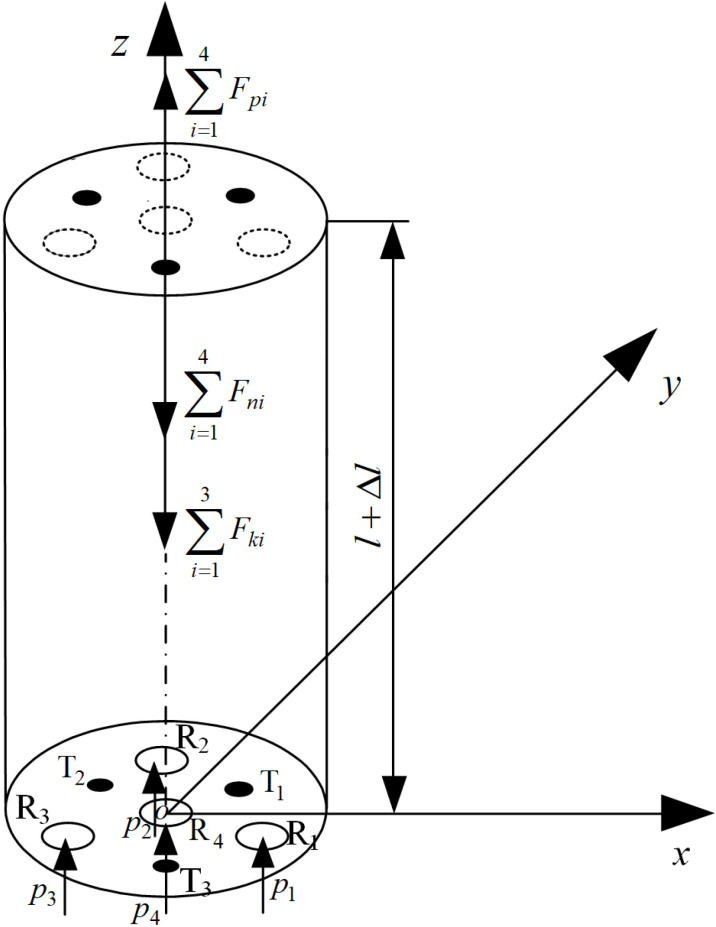
Analysis of joint axial deformation.

When the same compressed gas is injected into the four artificial muscles of the joint, the compressed gas generates axial force at the upper cover of the joint to drive the axial elongation of the joint. During the axial deformation process of the joint, the elastic skeleton and artificial muscle produce corresponding axial deformations, which impede the elongation of the joint and produce resistance forces, as shown in Fig 3. R_1_, R_2_, R_3_, R_4_ and T_1_, T_2_, T_3_ represent the four artificial muscles and three spring skeletons that make up the joint, respectively. The air pressure introduced into the artificial muscle is $p_{i}=p\;(i=1,\;2,\;3,\;4)$ , where 1, 2, 3 and 4 are the serial numbers of the artificial muscles.

According to the axial static equilibrium equation of the joint, it can be obtained that:


$$\sum_{i=1}^{4}F_{pi}=\sum_{i=1}^{4}F_{ni}+\sum_{i=1}^{3}F_{ki}$$ (1)

Where $F_{pi}$ is the driving force of artificial muscle, $F_{ni}$ is the impedance force of artificial muscle, $F_{ki}$ is the impedance force of the spring skeleton.

Under the condition of free elongation, the manufacturing error of artificial muscles is ignored. When the air pressure of each muscle is equal, that is $p_{1}=p_{2}=p_{3}=p_{4}$, the joint will be in an elongated state. The axial static equilibrium equation of joint can be written as:


$$\sum_{i=1}^{3}F_{pi}+F_{p4}=\sum_{i=1}^{3}F_{ni}+F_{n4}+\sum_{i=1}^{3}F_{ki}$$ (2)

At this time, the deformation situation of the joint is the same as the deformation law of a single artificial muscle. According to the deformation analysis of a single artificial muscle [24–25], the elongation of the joint can be derived as follows:


$$\begin{array}{c} \Delta l=\frac{4\pi p(3D_{2}^{2}+D_{2^{\prime }}^{2})l_{0}}{\pi E[3(D_{1}^{2}-D_{2}^{2})+(D_{1^{\prime }}^{2}-D_{2^{\prime }}^{2})]+12kl_{0}-4\pi p[3(D_{1}^{2}+D_{2}^{2})+(D_{1^{\prime }}^{2}+D_{2^{\prime }}^{2})]} \end{array}$$ (3)

Where Δl is the elongation of the joint, $l_{0}$ is the initial effective deformation length of the joint, E is the elastic modulus of the silicone tube, *k* is the spring stiffness, $D_{1}$ is the outer diameter of ${\text{R}}_{\text{1}}$, ${\text{R}}_{\text{2}}$ and ${\text{R}}_{\text{3}}$ artificial muscle silicone tubes, $D_{2}$ is the inner diameter of ${\text{R}}_{\text{1}}$, ${\text{R}}_{\text{2}}$ and ${\text{R}}_{\text{3}}$ artificial muscle silicone tubes before deformation, $D_{1^{\prime }}$ is the outer diameter of ${\text{R}}_{\text{4}}$ artificial muscle silicone tube, $D_{2^{\prime }}$ is the inner diameter of ${\text{R}}_{\text{4}}$ artificial muscle silicone tubes before deformation.

#### 3.1.2 Analysis of joint bending deformation.

**Fig 4:**
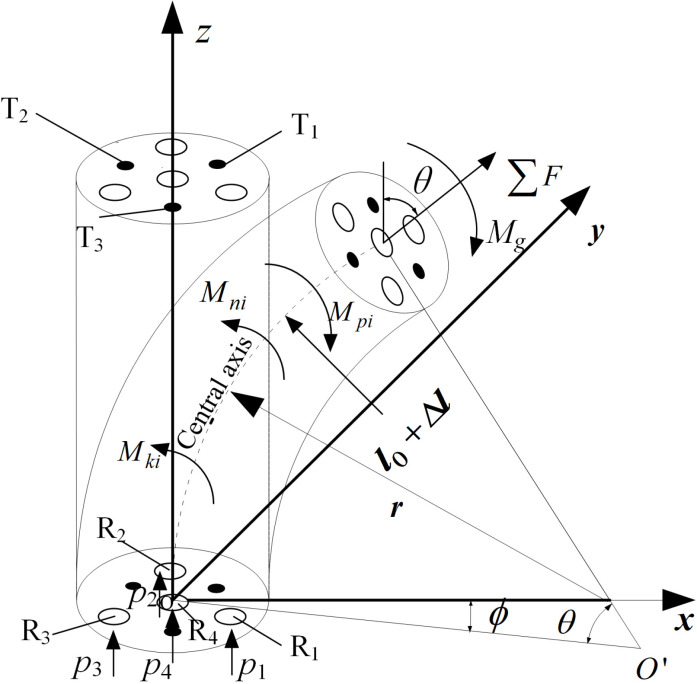
Analysis of joint bending deformation.

When artificial muscles${\text{R}}_{\text{1}}$, ${\text{R}}_{\text{2}}$ and ${\text{R}}_{\text{3}}$ are subjected to different air pressures, the joint is subjected to the combined action of driving moment$\sum M_{p_{i}}$, the spring impedance moment $\sum M_{k_{i}}$, and the silicone tube impedance moment$\sum M_{n_{i}}$.The direction of the resultant moment $M_{g}$ determines the bending direction of the joint, and the size of the resultant moment determines the size of the bending angle of the joint, as shown in Fig 4.

(1) Bending direction of the joint

After the compressed gas is introduced into the joint, the direction of the resultant moment received by the joint end is the same as the direction of the driving moment, and its expression is as follows:


$$\begin{array}{c} M_{g}=\sum_{i=1}^{3}M_{pi},\;i=1,\;2,\;3 \end{array}$$ (4)

The driving moment generated by a single artificial muscle can be expressed as follows:


$$\begin{array}{c} M_{pi}=p_{i}S_{i}l,\;i=\text{1},\text{ 2},\text{ 3} \end{array}$$ (5)

Where $S_{i}$ is the cross-sectional area of the inner cavity after deformation of artificial muscle, l  is the distance from the geometric center of the artificial muscle to the center of the joint.

**Fig 5:**
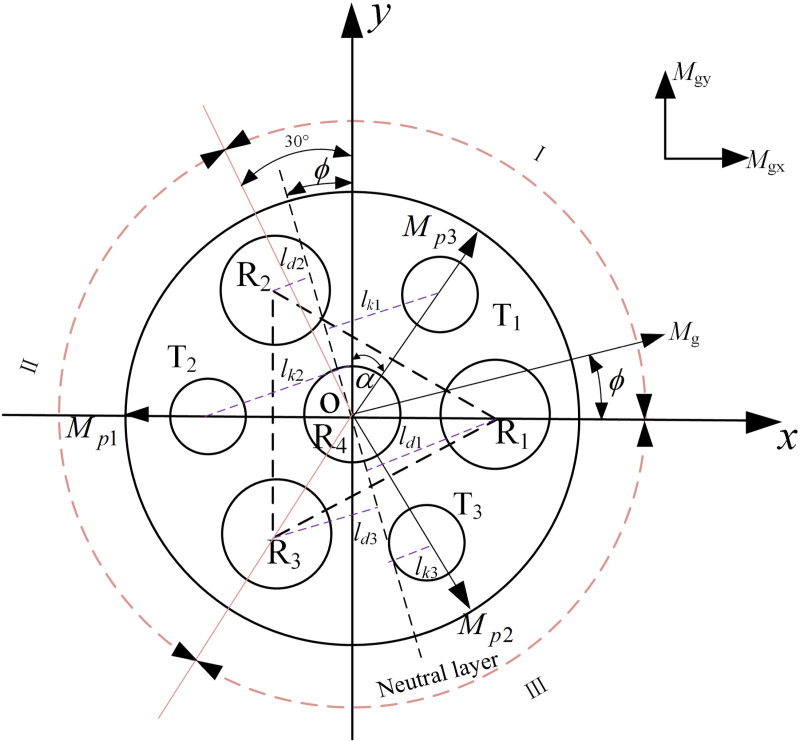
Distance from muscle and spring to neutral layer.


According to the moment analysis of the upper cover of the joint as shown in Fig 5, the bending direction of the joint can be expressed as follows:

$$\phi =arctan\frac{M_{gx}}{M_{gy}}$$ (6)

$$M_{gx}=(M_{p2}+M_{p3}sin\alpha -M_{p1}$$ (7)

$$M_{gy}=\text{(}M_{p3}-M_{p2}\text{)cos }\;\;\alpha \;\;\;$$ (8)

Where $M_{gx}$ is the component of the resultant moment on the *x*-axis, $M_{gy}$ is the component of the resultant moment on the *y*-axis, α=π/6 is the angle between the direction of the artificial muscle driving moment and the *y*-axis.

By substituting Equations (7) and (8) into Equation (6), the relationship between bending direction of the joint and the air pressure can be derived as follows:


$$\begin{array}{c} \phi =\text{arctan}\frac{\sqrt{3}\left(p_{3}S_{3}-p_{2}S_{2}\right)}{p_{2}S_{2}+p_{3}S_{3}-2p_{1}S_{1}} \end{array}$$ (9)

As can be seen from Equation (9), the bending direction of the joint depends on artificial muscles ${\text{R}}_{\text{1}}$, ${\text{R}}_{\text{2}}$ and ${\text{R}}_{\text{3}}$, and is independent of ${\text{R}}_{\text{4}}$. The reason is that the artificial muscle ${\text{R}}_{\text{4}}$ is located on the central axis of the joint, and only generates axial driving force to increase the axial elongation and deformation under the action of air pressure, but does not produce driving moment.

(2) Bending angle of the joint

The external constraint ring of the joint only serves as a constraint hinge, limiting the radial expansion of the internal silicone tube without generating impedance moment. According to the moment balance equation at the upper cover of the joint, it can be concluded that:


$$\sum_{i=1}^{3}M_{pi}=\sum_{i=1}^{3}M_{ki}+\sum_{i=1}^{4}M_{ni}$$ (10)

The joint bends along the neutral layer under the action of compressed gas, and the distance between the artificial muscle and the elastic skeleton and the neutral layer is shown in Fig 5. The artificial muscles and elastic skeleton are distributed in the same circumference. Because of the symmetrical distribution of artificial muscles, the bending of joint is the same in the three equally divided regions I, II, and III. The bending states of each muscle and elastic skeleton within the bending direction of 0 ~ 120° in region I are as follows: When 0≤φ≤30∘, artificial muscles${\text{R}}_{\text{2}}$, ${\text{R}}_{\text{3}}$ and ${\text{R}}_{\text{4}}$ are in an extended state, while ${\text{R}}_{\text{1}}$ is compressed, elastic skeleton ${\text{T}}_{\text{2}}$ is in an extended state, while ${\text{T}}_{1}$ and${\text{T}}_{3}$ are compressed; When 30∘<φ≤90∘, artificial muscles ${\text{R}}_{\text{3}}$ and ${\text{R}}_{\text{4}}$ are in an extended state, while ${\text{R}}_{\text{1}}$ and ${\text{R}}_{\text{2}}$ are compressed, elastic skeletons ${\text{T}}_{\text{2}}$ and ${\text{T}}_{3}$ are in an extended state, while ${\text{T}}_{1}$ is compressed; When 90∘<φ≤120∘, artificial muscles ${\text{R}}_{\text{1}}$, ${\text{R}}_{\text{2}}$ and ${\text{R}}_{\text{4}}$ are in an extended state, while ${\text{R}}_{\text{3}}$ is compressed, elastic skeleton ${\text{T}}_{3}$ is in an extended state, while ${\text{T}}_{1}$ and ${\text{T}}_{\text{2}}$ are compressed.

a) Moment analysis at 0≤φ≤30∘

The driving moment at the upper cover of the joint is the normal component of the driving moment of each artificial muscle in the neutral layer. The expression can be obtained as follows:


$$\sum_{i=1}^{3}M_{pi}=p_{2}S_{2}l_{d2}+p_{3}S_{3}l_{d3}-p_{1}S_{1}l_{d1}$$ (11)

According to the geometric relationship in Fig 5, it can be seen that:


$$\begin{array}{c} \left\{ \begin{array}{cc} & l_{d1}=l\cos\phi \\ & l_{d2}=l\sin(\alpha -\phi )\\ & l_{d3}=l\sin(\alpha +\phi ) \end{array}\right. \end{array}$$ (12)

Where $l_{d1}$, $l_{d2}$ and $l_{d3}$ are the distances from the center of the artificial muscle to the neutral layer of joint bending.

Due to the approximate circular arc shape of joint bending, the deformation coordination equation of the spring under joint bending can be obtained as follows:


$$\left\{ \begin{array}{cc} @\;\mathrm{-8pt}ll & \Delta l_{1}^{\prime }=\Delta l-l_{k1}\theta \\ & \Delta l_{2}^{\prime }=\Delta l+l_{k2}\theta \\ & \Delta l_{3}^{\prime }=\Delta l-l_{k3}\theta \end{array}\right.$$ (13)

Where $l_{k1}$, $l_{k2}$ and $l_{k3}$ are the distances from the center of the spring to the neutral layer of joint bending. As can be seen from the geometric relationship in Fig 5: $l_{k1}$ =$l_{d3}$,$\;l_{k2}$ = $l_{d1}$,$\;l_{k3}$ =$l_{d2}$.

When the elastic skeleton resists the bending deformation of the joint, apart from the bending moment generated during body-bending, a coupling moment also occurs when bending around the joint’s bending center. Based on the deformation coordination condition, we assume that the bending moments of the elastic skeleton follow the principle of linear superposition. Additionally, the coupling moment generated in the axial direction of the elastic skeleton is taken into account. Thus, the total impedance moment of the elastic skeleton can be derived as follows:


$$\sum_{i=1}^{3}M_{ki}=3M_{k}+k(\Delta ll_{2}^{\prime }-\Delta ll_{1}^{\prime }-\Delta ll_{3}^{\prime })$$ (14)

According to the pure bending moment deformation formula of the cylindrical helical spring, considering the influence of prestress on the bending deformation of the spring [26], the bending moment of the cylindrical helical spring can be obtained as follows:


$$\begin{array}{c} M_{k}=\frac{E_{1}d^{4}}{32Dn(2+\mu )}\theta +M_{0} \end{array}$$ (15)

Where $E_{1}$ is the elastic modulus of the spring, μ is Poisson’s ratio, D is the middle diameter of the spring, d is the steel wire diameter of spring, n is the number of effective turns of the spring, $\begin{array}{c} M_{0} \end{array}$ is the initial moment generated by spring prestress.

The deformation coordination equation of silicone tube under joint bending is as follows:


$$\left\{ \begin{array}{cc} @\;\mathrm{-8pt}ll & \Delta l_{1}=\Delta l-l_{1}\sin\theta \\ & \Delta l_{2}=\Delta l+l_{2}\sin\theta \\ & \Delta l_{3}=\Delta l+l_{3}\sin\theta \\ & \Delta l_{4}=\Delta l \end{array}\right.$$ (16)

The impedance moment generated by the bending of silicone tube is the same as that of the elastic skeleton, including the impedance moment of the body and the coupling moment. The total impedance moment of silicone tube can be obtained as follows:


$$\sum_{i=1}^{4}M_{ni}=4M_{n}+k_{g}(\Delta ll_{2}+\Delta ll_{3}-\Delta ll_{1})$$ (17)

Where $M_{n}$ is the impedance moment generated when the silicone tube is bent around its own body, and its expression is as follows [24]: $M_{n}=\frac{\pi E(D_{1}^{2}-D_{2}^{2})[(D_{1}^{2}+D_{2}^{2})l_{0}^{2}+2D_{1}^{2}l_{0}\Delta l]}{64{\left(l_{0}+\Delta l\right)}^{3}}\theta$,$k_{g}=\frac{F_{n}}{\Delta l}=\frac{\pi E}{4}(D_{1}^{2}-D_{2}^{2})l_{0}\frac{l_{0}^{2}-\Delta l^{2}}{{\left(l_{0}+\Delta l\right)}^{4}}$ is the axial stiffness of the silicone tube.

b) Moment analysis at 30∘<φ≤90∘

When the bending direction is in this interval, artificial muscles ${\text{R}}_{\text{3}}$ and ${\text{R}}_{\text{4}}$ are in an extended state, while ${\text{R}}_{\text{1}}$ and ${\text{R}}_{\text{2}}$ are compressed, elastic skeletons ${\text{T}}_{\text{2}}$ and ${\text{T}}_{3}$ are in an extended state, while ${\text{T}}_{1}$ is compressed.

The driving moment of the joint is as follows:


$$\sum_{i=1}^{3}M_{pi}=p_{3}S_{3}l_{d3}-p_{2}S_{2}l_{d2}-p_{1}S_{1}l_{d1}$$ (18)

In the above formula, $l_{d1}=l\cos\varphi$,$l_{d2}=l\sin(\varphi -\alpha )$,$l_{d3}=l\sin(\varphi +\alpha )$.

The deformation coordination equations of elastic and silicone tube under joint bending deformation are as follows:


$$\left\{ \begin{array}{cc} @\;\mathrm{-8pt}ll & \Delta l_{1}^{\prime }=\Delta l-l_{k1}\theta \\ & \Delta l_{2}^{\prime }=\Delta l+l_{k2}\theta \\ & \Delta l_{3}^{\prime }=\Delta l+l_{k3}\theta \end{array}\right.\;\mathrm{-5pt},\;\;\;\left\{ \begin{array}{cc} @\;\mathrm{-8pt}ll & \Delta l_{1}=\Delta l-l_{1}\sin\theta \\ & \Delta l_{2}=\Delta l-l_{2}\sin\theta \\ & \Delta l_{3}=\Delta l+l_{3}\sin\theta \\ & \Delta l_{4}=\Delta l \end{array}\right.$$ (19)

The total impedance moments of the elastic components and the silicone tubes are as follows:


$$\sum_{i=1}^{3}M_{ki}=\frac{3E_{1}d_{1}^{4}}{32Dn(2+\mu )}+k(\Delta ll_{2}^{\prime }+\Delta ll_{3}^{\prime }-\Delta ll_{1}^{\prime })$$ (20)

$$\sum_{i=1}^{4}M_{ni}=\frac{\pi E(D_{1}^{2}-D_{2}^{2}lbrack(D_{1}^{2}-D_{2}^{2})l_{0}^{2}+2D_{1}^{2}l_{0}\Delta l]}{16(l_{0}+\Delta l{)}^{3}}\theta +k_{g}(\Delta ll_{3}-\Delta ll_{2}-\Delta ll_{1})$$ (21)

c) Moment analysis at 90∘<φ≤120∘

When the bending direction is in this interval, artificial muscles ${\text{R}}_{\text{1}}$, ${\text{R}}_{\text{2}}$ and ${\text{R}}_{\text{4}}$ are in an extended state, while ${\text{R}}_{\text{3}}$ is compressed, elastic skeleton ${\text{T}}_{3}$ is in an extended state, while${\text{T}}_{1}$ and ${\text{T}}_{\text{2}}$ are compressed.

The driving moment of the joint is as follows:


$$\sum_{i=1}^{3}M_{pi}=p_{1}S_{1}l_{d1}+p_{2}S_{2}l_{d2}-p_{3}S_{3}l_{d3}$$ (22)

In the above formula, $l_{d1}=l\text{sin(}\varphi -\pi /2)\;$,$l_{d2}=l\sin(\pi -\varphi +\alpha )$,$l_{d3}=l\sin(\pi -\varphi -\alpha )$.

The deformation coordination equations of elastic and silicone tube under joint bending deformation are as follows:


$$\left\{ \begin{array}{cc} @\;\mathrm{-8pt}ll & \Delta l_{1}^{\prime }=\Delta l-l_{k1}\theta \\ & \Delta l_{2}^{\prime }=\Delta l-l_{k2}\theta \\ & \Delta l_{3}^{\prime }=\Delta l+l_{k3}\theta \end{array}\right.\;\mathrm{-5pt},\;\;\;\left\{ \begin{array}{cc} @\;\mathrm{-8pt}ll & \Delta l_{1}=\Delta l+l_{1}\sin\theta \\ & \Delta l_{2}=\Delta l+l_{2}\sin\theta \\ & \Delta l_{3}=\Delta l-l_{3}\sin\theta \\ & \Delta l_{4}=\Delta l \end{array}\right.$$ (23)

The total impedance moments of elastic and silicone tubes are as follows:


$$\sum_{i=1}^{3}M_{ki}=\frac{3E_{1}d_{1}^{4}}{32Dn(2+\mu )}+k(\Delta ll_{3}^{\prime }-\Delta ll_{1}^{\prime }-\Delta ll_{2}^{\prime })$$ (24)

$$\sum_{i=1}^{4}M_{ni}=\frac{\pi E(D_{1}^{2}-D_{2}^{2}lbrack(D_{1}^{2}-D_{2}^{2})l_{0}^{2}+2D_{1}^{2}l_{0}\Delta l]}{16(l_{0}+\Delta l{)}^{3}}\theta +k_{g}(\Delta ll_{1}+\Delta ll_{2}-\Delta ll_{3})$$ (25)

By substituting Equations (11) to (25) into Equation (10), the bending angle of the joint in region I can be derived.

### 3.2 Theoretical analysis of gripper

The designed gripper is composed of a dual-muscle-driven pneumatic unidirectional bending joint and an anchor connected in series, and its mechanical performance depends on the combined action of the two. Theoretical modeling and experimental research were conducted on the bending angle and output force of joint in the early stage [27–28].

**Fig 6:**
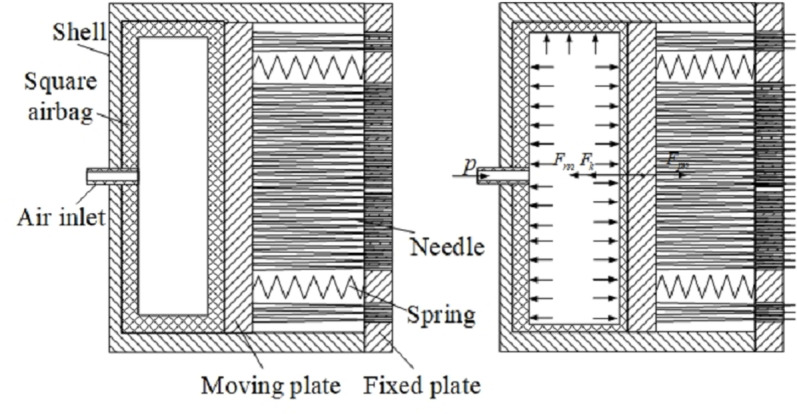
The anchor working mechanism.

The working principle of the anchor is shown in Fig 6. After applying compressed gas, the square airbag is compressed and expanded to push the moving plate, which drives the needle to extend out of the fixed plate and contact the tree surface, thereby increasing the anchoring force between the robot legs and the tree surface, and improving the robot’s load capacity.

According to the working principle and force analysis of the anchor, the static equilibrium equation acting on the moving plate is as follows:


$$F_{pn}=F_{nn}+2F_{k}$$ (26)

Where $F_{pn}$ is the driving force generated by the air pressure acting on the square airbag, $F_{nn}$ is the resistance generated by the deformation of the square airbag itself, and which is related to the elastic modulus and deformation amount of the airbag. $F_{k}$ is the impedance force generated by the reset spring.

When the anchor is in contact with the tree surface, the needle extension is limited. Therefore, the impedance force generated by the deformation of the airbag and spring can be ignored. The output force of the anchor mainly depends on the airbag pressure p and the cross-sectional area of the square airbag cavity S, which is expressed as follows:


$$F=F_{pn}-Fn_{n}-F_{k}=cpS$$ (27)

Where c is the correction factor, mainly to correct the nonlinear change of the cross-sectional area of the airbag with its elongation.

The dual-muscle-driven pneumatic unidirectional bending joint exhibits distinct characteristics of large deformation and non-linearity. Once the anchor makes contact with the tree surface, the free deformation of the joint becomes restricted. In the event that the air pressure persists in increasing, the joint will display irregular passive deformation, thereby heightening the complexity of theoretical modeling for the gripper. Consequently, this paper will conduct experimental investigations on the clamping force and load capacity of the leg by means of experimental method.

## 4 Gait planning of robot

By adjusting the ventilation combinations and pressure gradient of each joint, and coordinating the alternating drives of the waist and legs, the robot can achieve gaits like climbing up, climbing down, and crossing obstacles on the tree. Among these gaits, climbing up is the process where the robot climbs upward along the trunk to reach close to the treetop, while climbing down is the process where the robot climbs downward along the trunk to approach the ground. Crossing obstacles on the tree is the process of avoiding obstacles such as branches and burls during climbing.

### 4.1 The climbing-up gait

**Fig 7:**
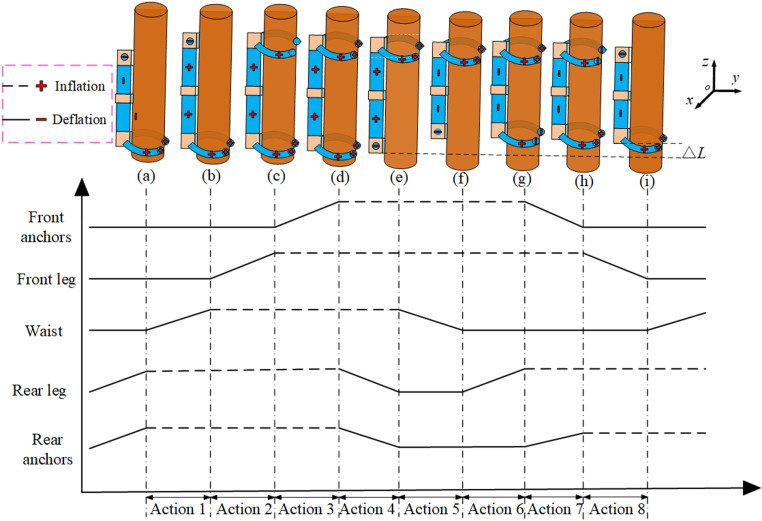
The gait and timing diagram for climbing-up.

As illustrated in Fig 7, compressed gas is injected into each joint according to the timing diagram, and the robot can climb upward along the trunk. To prevent the robot from falling under the action of its own weight and load, the rear leg and rear anchors (that is, two at the ends of the rear leg and the abdominal anchor, a total of three) are pressurized to encircle and clamp the trunk as the initial state. Next, the specific gait for climbing upward is divided into the following eight steps: ① Inject compressed gas of the same pressure into the upper and lower waist simultaneously. Then, the robot extends upwards along the tree trunk. ② When the elongation reaches a stable state, the robot’s front leg is inflated to tightly clamp the trunk. ③ Pressurize the front anchors (that is, two at the ends of the front leg and one abdominal anchor, three in total), and the needles stick out and thrust into the tree surface. ④ Depressurize the rear leg and rear anchors to release the trunk. ⑤ Depressurize the upper and lower waist simultaneously. Under the elastic effect of the springs and silicone tubes at the waist, the robot rebounds by a lengthΔL(that is, the step length of one upward tree-climbing cycle). ⑥ When the rebound is over, pressurize the robot’s rear leg to tightly clamp the trunk. ⑦ Pressurize the rear anchors, and the needles stick out and thrust into the tree surface. ⑧ Depressurize the front leg and front anchors to release the trunk.

### 4.2 The climbing-down gait

**Fig 8:**
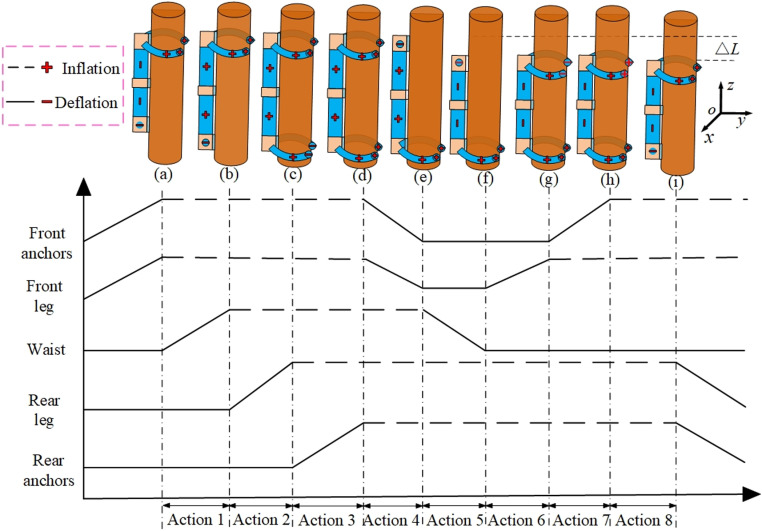
The gait and timing diagram for climbing-down.

As illustrated in Fig 8, the climbing-down gait is similar to the climbing-up gait. To prevent the robot from falling under the action of its own weight and load, the front leg and front anchors are pressurized to encircle and clamp the trunk as the initial state. Next, the specific gait for climbing down trees is divided into the following eight steps: ① Inject compressed gas of the same pressure into the upper and lower waist simultaneously. The robot then extends downward along the tree trunk. ② When the elongation reaches a stable state, the rear leg is pressurized to tightly clamp the trunk. ③ Pressurize the rear anchors, and the needles stick out and thrust into the tree surface. ④ Depressurize the front leg and front anchors to release the trunk. ⑤ Depressurize the upper and lower waist simultaneously. Under the elastic effect of the springs and silicone tubes at the waist, the robot rebounds by a lengthΔL.ΔLis the step distance for climbing up a tree in one cycle. ⑥ When the rebound is over, pressurize the front leg to tightly clamp the trunk. ⑦ Pressurize the front anchors, and the needles stick out and thrust into the tree surface. ⑧ Depressurize the rear leg and rear anchors to release the trunk.

### 4.3 The gait of crossing obstacles on trees

**Fig 9:**
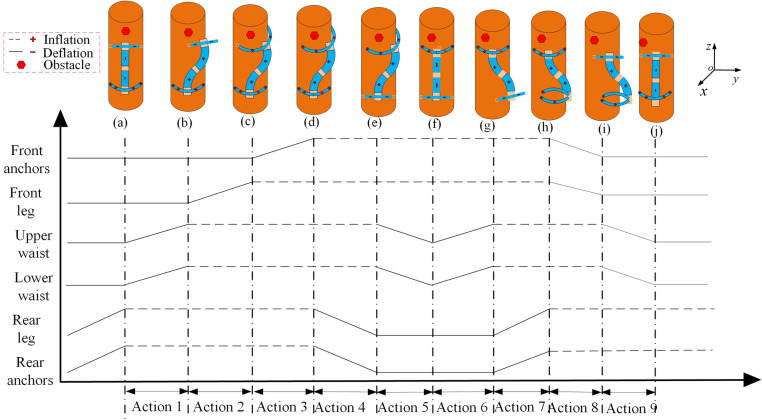
The gait and timing diagram for crossing obstacles on trees.

To avoid obstacles such as branches or burls on trees, the gait of crossing obstacles on trees and the ventilation timing sequence of each joint of the robot under this gait are meticulously designed, as illustrated in Fig 9. With the robot initially positioned beneath the obstacle, the rear leg and rear anchors are pressurized to encircle and firmly clamp the trunk. Subsequently, the specific climbing gait can be dissected into the following nine steps: ① Inject different pressures into the four muscles at the robot’s lower waist. This prompts the robot to bend inward towards the tree trunk, enabling its upper torso to avert obstacles at an angle. Subsequently, introduce varying pressures to the four muscles of the upper waist. The upper waist bends towards the direction of the trunk at an angle, making the upper torso of the robot parallel to the tree trunk and closely attached to the surface of the tree trunk, thus securing a suitable encircling position for the front leg. ② Pressurize the robot’s front legs to tightly clamp the trunk. ③ Exert pressure on the front anchors, making the needles protrude and thrust into the tree surface. ④ Depressurize the rear leg and rear anchors to release the trunk. ⑤ Simultaneously relieve pressure on the upper and lower waists. This allows the waist and rear leg to rebound to the plane of the front leg, causing the robot to rotate as a whole by a certain angle. ⑥ Adjust the pressure of the artificial muscles in the upper and lower waists. Specifically, utilize the upper waist to modulate the lower torso’s position and the lower waist to adapt the rear leg’s posture into a suitable encircling state. ⑦ Pressurize the rear leg and rear anchors to tightly clutch the trunk. ⑧ Depressurize the front leg and front anchors to release the trunk. ⑨ Simultaneously release the pressure in the upper and lower waists, permitting the robot’s torso to rebound. Subsequently, the robot rotates as a whole by an additional angle.

## 5 Experiment and analysis

**Fig 10:**
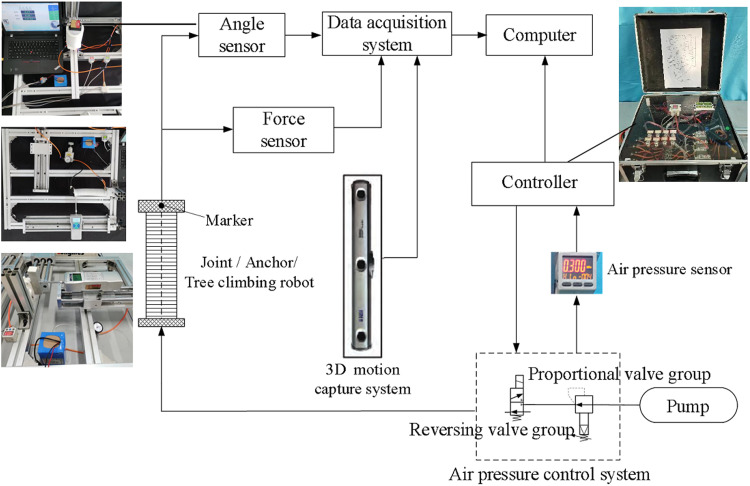
Integrated experimental system for joints and robot.

As the key components of the tree-climbing robot, the mechanical properties of the pneumatic flexible joint and anchor directly affect the motion performance of the robot, and the specific structural parameters are shown in Tables 1–3. Using the integrated experimental system for joints and robots shown in Fig 10, experiments were carried out respectively on the joint deformation characteristics (including elongation, bending angle and output force), the anchoring force of the anchor, the clamping performance of the leg and the motion performance of the robot.

**Table 1:** Structure parameters of the enhanced pneumatic flexible spatial bending joint.

Parameter	Numerical value	Unit
Initial inner diameter of silicone tubes in muscles ${\text{R}}_{\text{1}}$, ${\text{R}}_{\text{2}}$ and ${\text{R}}_{\text{3}}$	20	mm
Initial outer diameter of silicone tubes in muscles ${\text{R}}_{\text{1}}$, ${\text{R}}_{\text{2}}$ and ${\text{R}}_{\text{3}}$	24	mm
Initial inner diameter of silicone tube in muscle ${\text{R}}_{\text{4}}$	12	mm
Initial outer diameter of silicone tube in muscle ${\text{R}}_{\text{4}}$	16	mm
Effective deformation length of the joint	120	mm
Total length of the joint	140	mm
Elastic modulus of the silicone tube	2.277	MPa
Diameter of spring steel wire	2.2	mm
Pitch diameter of the spring	16	mm
Pitch of the spring	6	mm
Number of effective turns of the spring	23	
Diameter of the spring distribution circle	25	mm
Diameter of the constraint ring	80	mm
Thickness of the constraint ring	2	mm

**Table 2:** Structure parameters of the pneumatic unidirectional bending joint.

Parameter	Numerical value	Unit
Initial inner diameter of the silicone tube	20	mm
Initial outer diameter of the silicone tube	24	mm
Elastic modulus of the silicone tube	2.277	MPa
Effective deformation length of the joint	150	mm
Total length of the joint	180	mm
Length of the spring steel plate	170	mm
Width of the spring steel plate	30	mm
Thickness of the spring steel plate	0.25	mm
Elastic modulus of spring steel plate	206	GPa
Thickness of the constraint ring	2	mm

**Table 3:** Structure parameters of the Anchor.

parameter	Numerical value	Unit
Length of the anchor	50	mm
Width of the anchor	30	mm
Height of the anchor	40	mm
Length of the inner cavity of the square airbag	15	mm
Width of the inner cavity of the square airbag	12	mm
Length of the needle puncture	30	mm

### 5.1 Experimental analysis of joints

#### 5.1.1 Experimental study on enhanced pneumatic flexible spatial bending joint.

(1) Elongation and driving force of the joint

**Fig 11:**
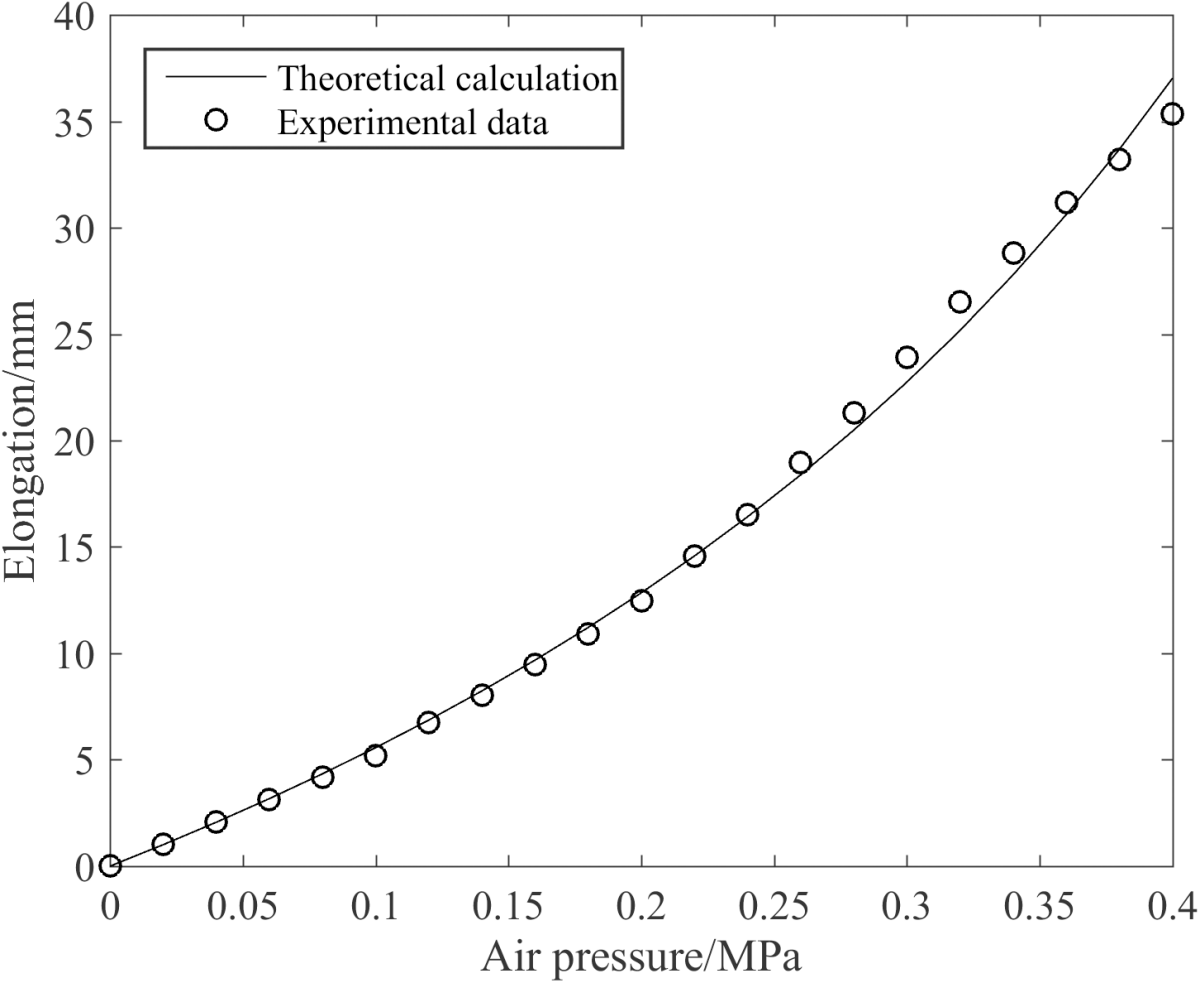
Elongation variation curve of the joint with respect to air pressure.

The elongation under the action of different air pressures was measured by using the 3D motion capture system, as depicted in Fig 11. It can be seen from Fig 11 that the theoretical calculation of the joint elongation is consistent with the experimental data trend, and the maximum error between the two is 7.10%, which shows that the theoretical formula can truly reflect the deformation law. The elongation increases nonlinearly with the increase of air pressure. This phenomenon can be ascribed to the fact that, upon the deformation of muscles under different pressure magnitudes, the cross-sectional areas of the inner cavities of the silicone tubes vary, thereby leading to inconsistent muscle driving forces.

**Fig 12:**
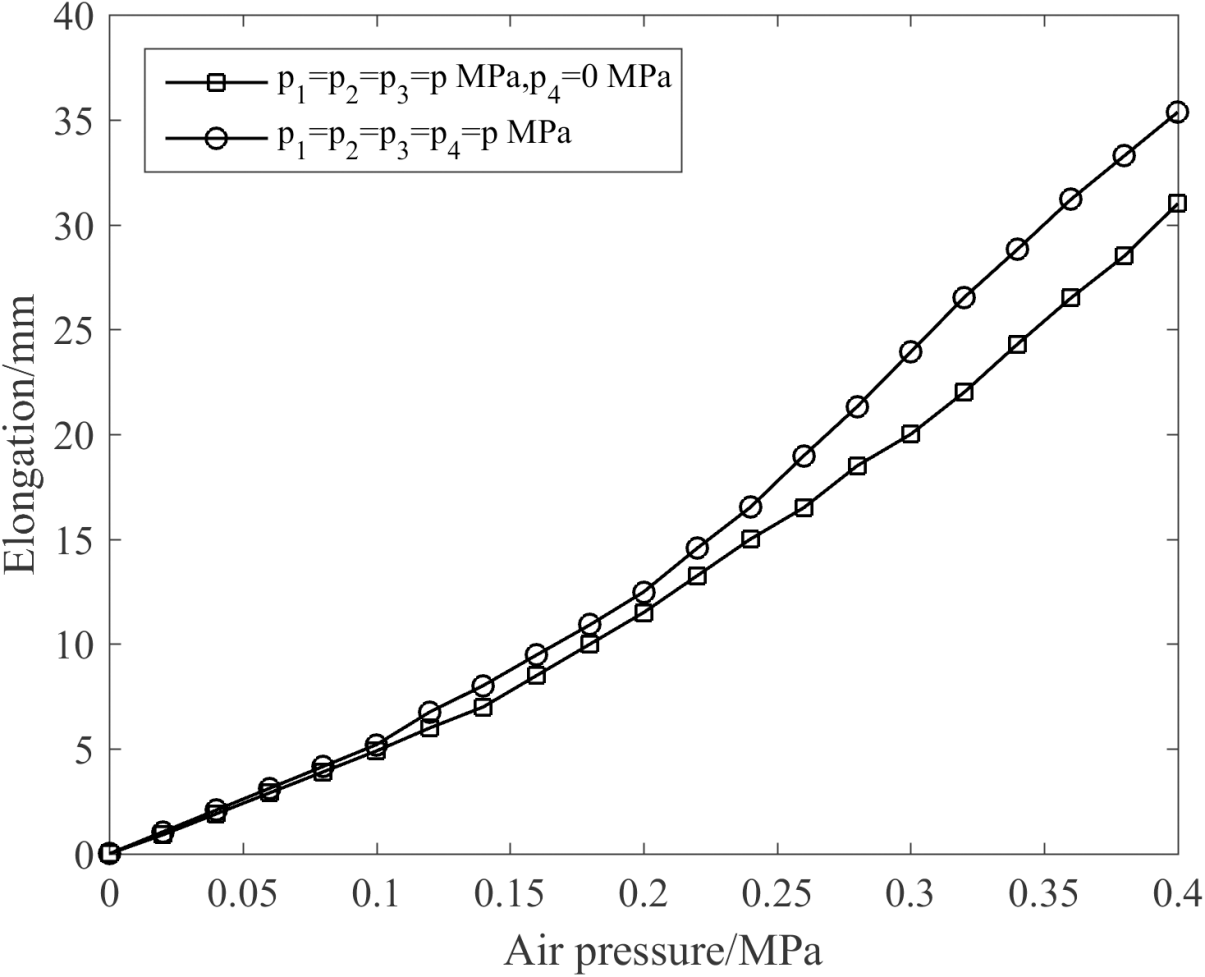
Influence of the enhanced muscle on joint elongation performance.

Fig 12 illustrates the effect of the enhanced muscle on joint elongation. As can be discerned from Fig 12, when the enhanced muscle is pressurized, it exerts an amplifying influence on the joint elongation. In the enhanced state, the joint elongation reaches 35.4 mm at 0.4 MPa, representing a 14.2% increment compared to the non-enhanced state.

**Fig 13:**
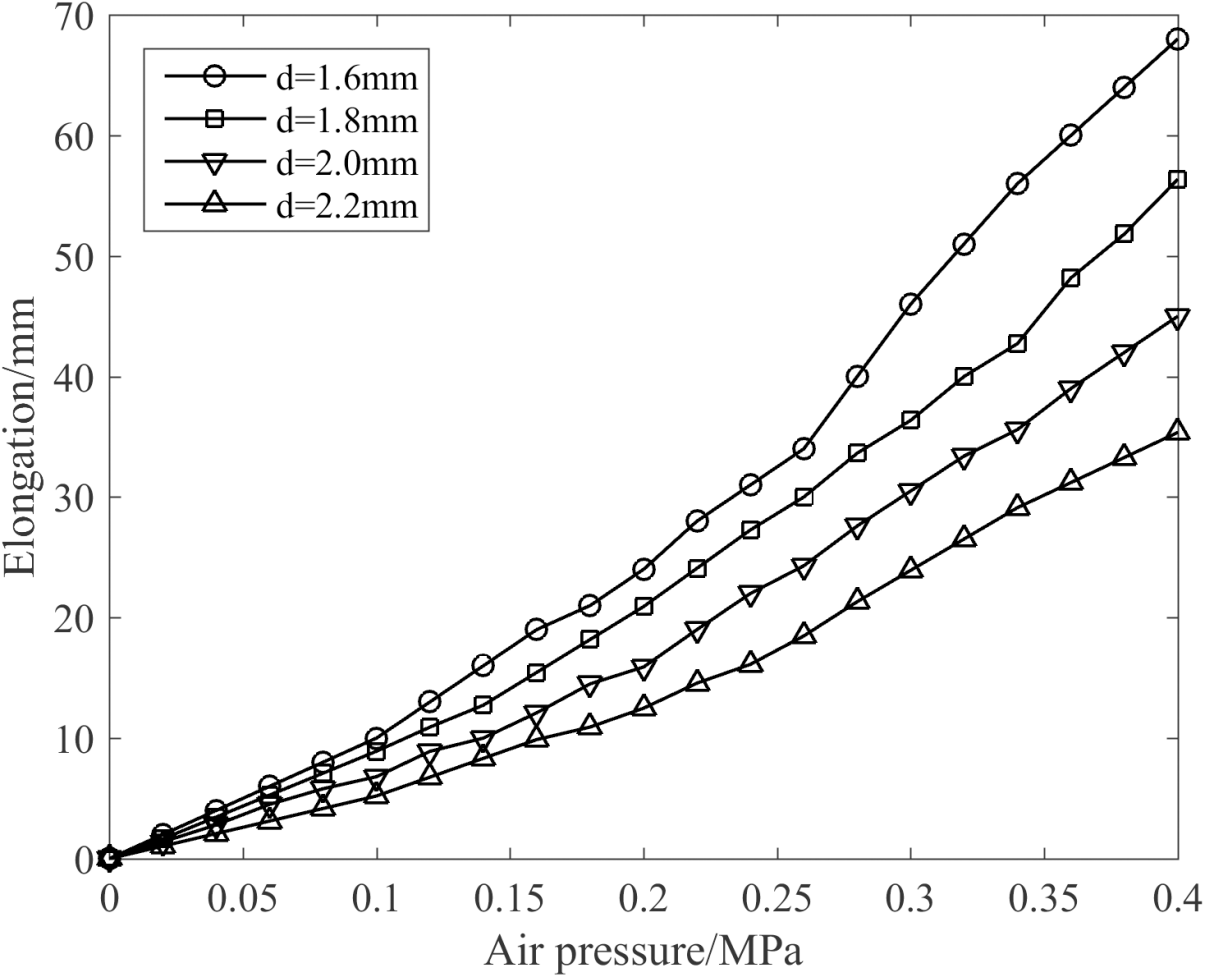
Influence of diverse wire diameters on the joint’s elongation.

Fig 13 shows the effect of different spring wire diameters on joint elongation when four artificial muscles are simultaneously pressurized with an identical air pressure. As observable from Fig 13, as the wire diameter increases, the axial stiffness of the joint rises, which in turn causes a progressive decline in joint elongation. At 0.4 MPa, with a wire diameter of 1.6 mm, and the maximum elongation of the joint reaches 68 mm.

**Fig 14:**
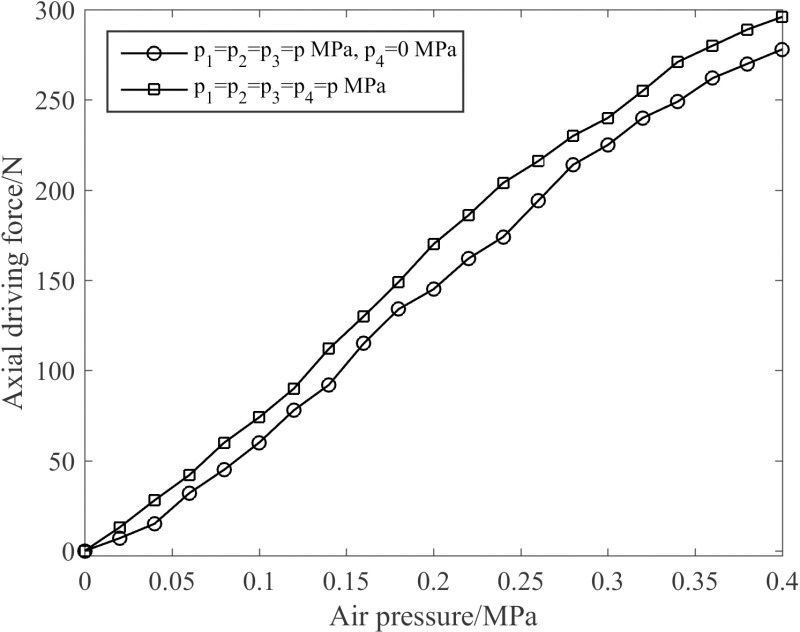
Influence of the enhanced muscle on the axial driving force of the joint.

The axial driving force of the joint under different air pressures was gauged by means of a digital display push-pull force gauge. Fig 14 presents the influence of the enhanced muscle on the axial driving force when the joint’s deformation is constrained at the initial position (that is, the elongation is 0). As can be seen from Fig 14, the joint axial driving force gradually increases with the increase of air pressure. When the enhanced muscle is pressurized, it exerts an amplifying effect on the axial driving force of the joint. At 0.4 MPa, the axial driving force of the joint reaches 296 N, signifying an increment of 6.5%.

**Fig 15:**
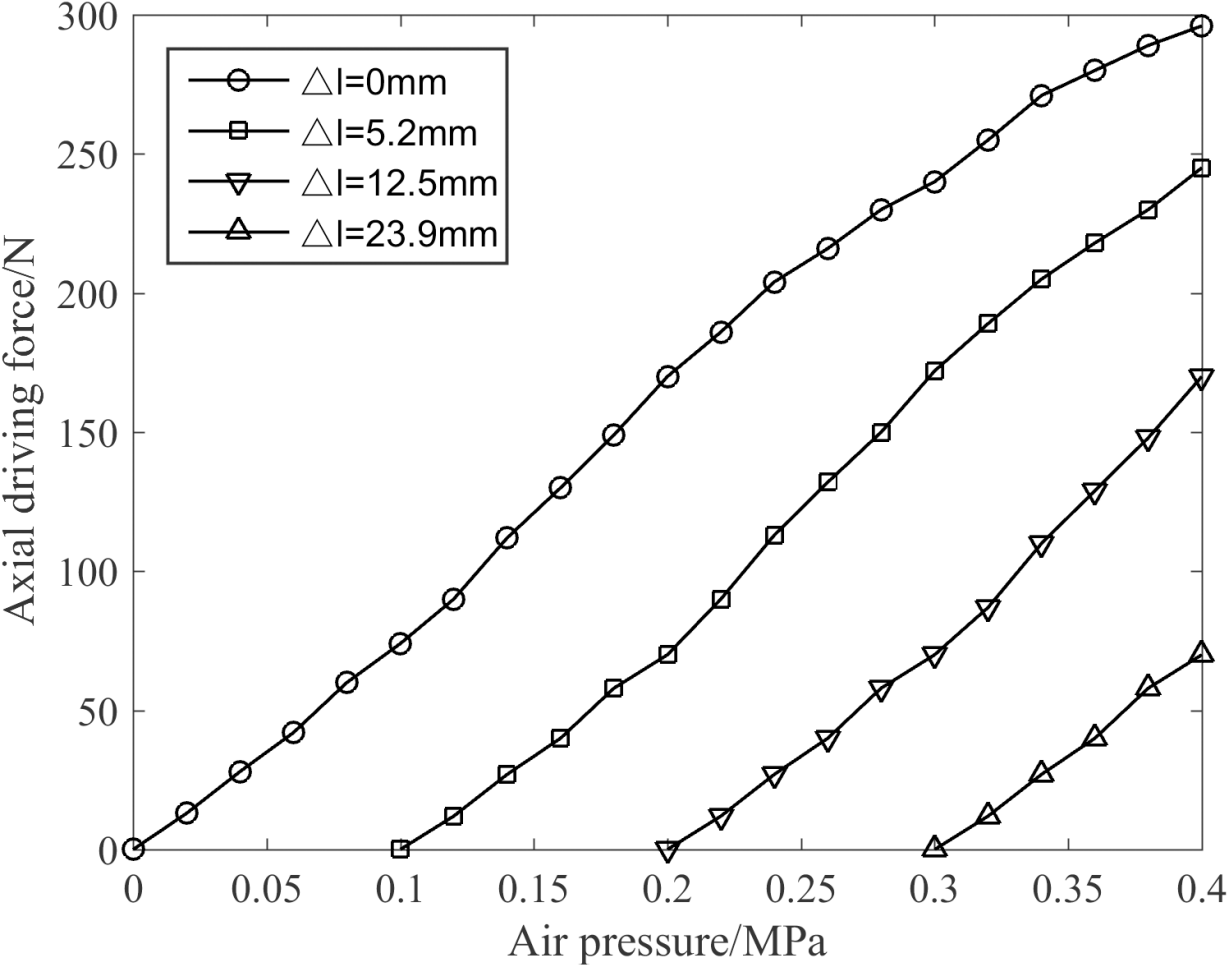
Characteristics of the axial driving force of the joint.

Fig 15 displays the comparison curve of joint driving force under varying elongation limitations when all four muscles are actuated simultaneously. As depicted in Fig 15, when the air pressure remains constant, the axial driving force of the joint is inversely proportional to the elongation. When the elongation is constant, the axial driving force of the joint is proportional to the pressure value. Consequently, when $p_{1}=p_{2}=p_{3}=p_{4}=0.4\text{ MPa}$ and the elongation is 0 mm, the axial driving force is at its maximum.

(2) Bending direction of the joint

**Fig 16:**
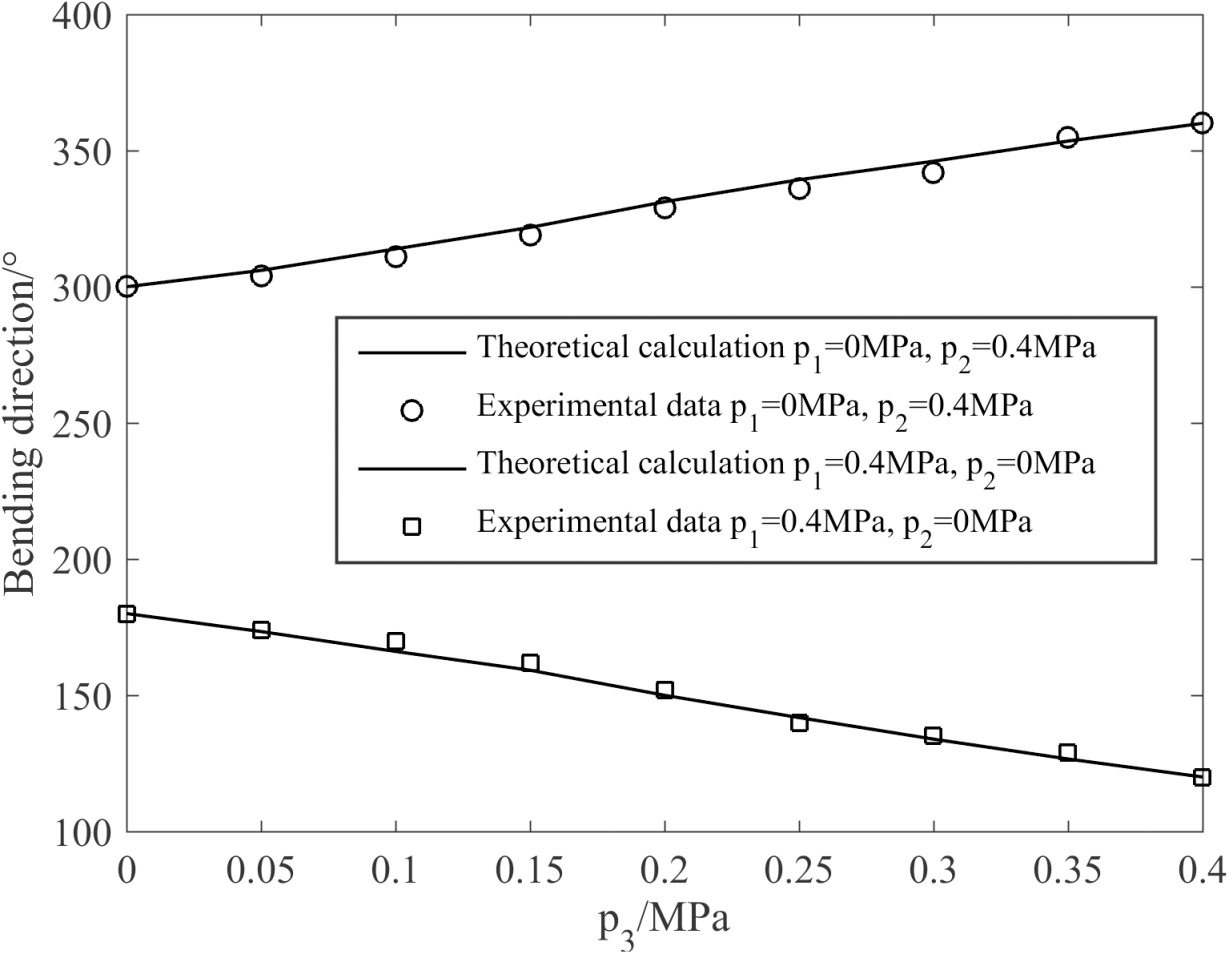
Variation curves of the joint bending direction in response to air pressure.

Substituting the joint parameters enumerated in Table 1 into Equation (9), the variation of the joint bending direction with air pressure as depicted in Fig 16. It can be seen from Fig 16 that the changing trends of the theoretical calculation and the experimental data regarding the joint bending direction are consistent and in good agreement, indicating that the established theoretical model can truly reflect the variation law of the joint bending direction with air pressure. Specifically, when the air pressures of $p_{1}$ and $p_{2}$ remain constant, the joint’s bending direction will correspondingly increase or decrease in tandem with the air pressure of $p_{3}$.

**Fig 17:**
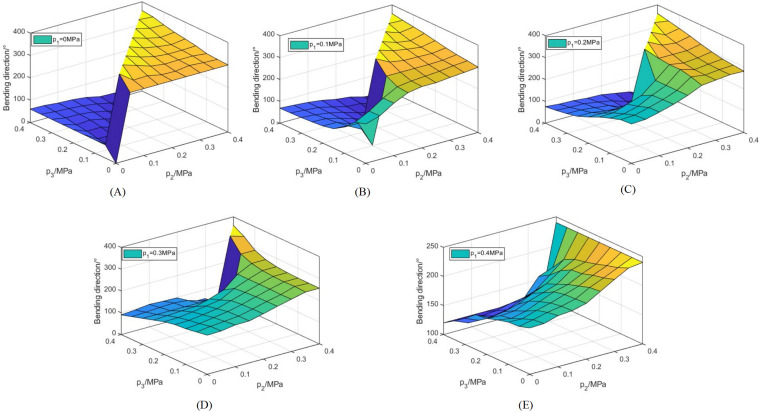
Variation curves of the joint bending direction in response to air pressure. (A) $p_{1}=0\text{MPa}$. (B) $p_{1}=0.1\text{MPa}$. (C) $p_{1}=0.2\text{MPa}$. (D) $p_{1}=0.3\text{MPa}$. (E) $p_{1}=0.4\text{MPa}$.

As depicted in Fig 17, when the air pressure of one artificial muscle is maintained at a constant level and the air pressures of the other two artificial muscles are precisely adjusted, the joint can be controlled to bend in any arbitrary direction within the angular range of 0° to 360°.

(3) Bending angle of the joint

**Fig 18:**
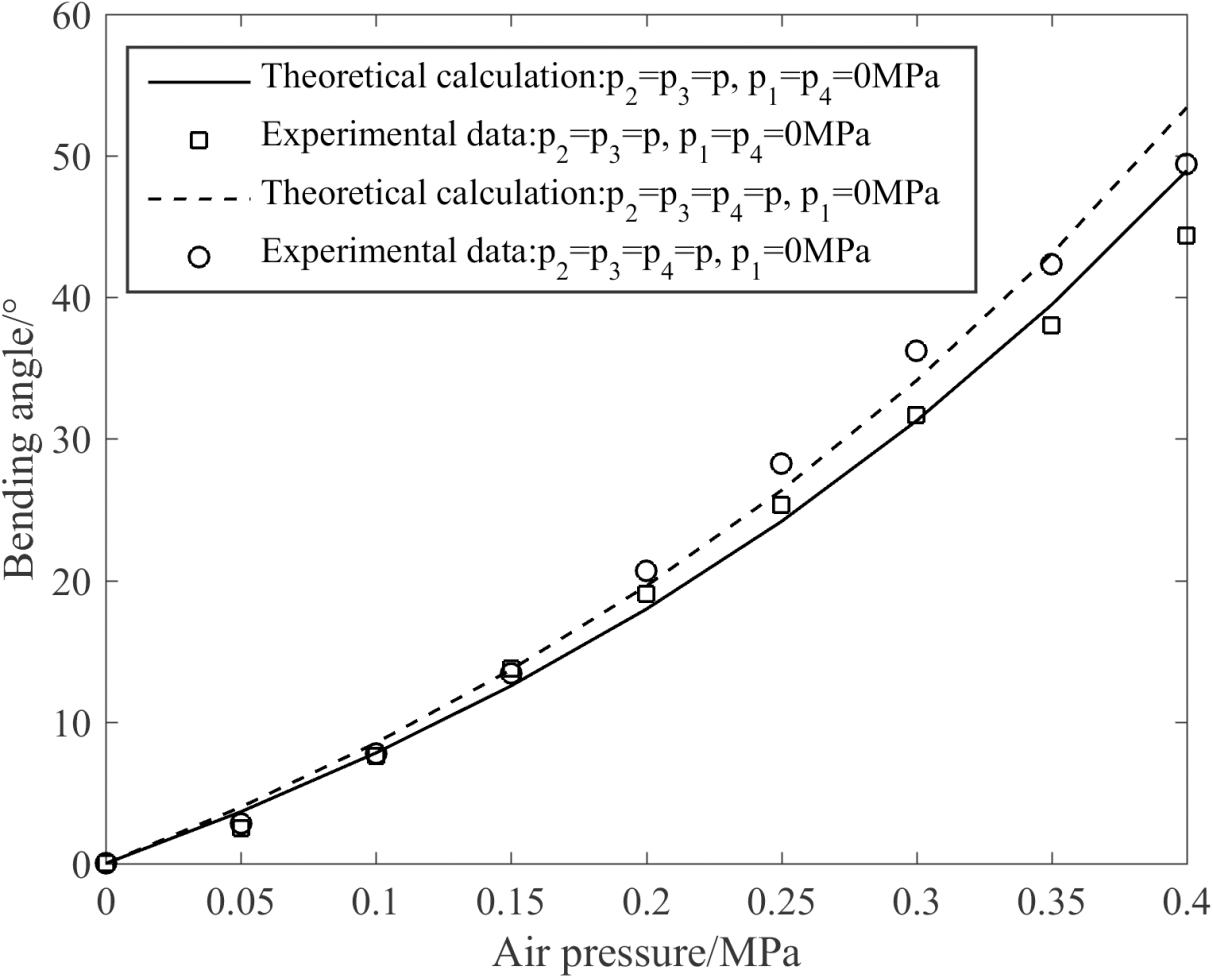
Variation curves of the joint’s bending angle with air pressure.

Fig 18 presents the various curves of the joint’s bending angle with air pressure. As can be discerned from Fig 18, the trends exhibited by the theoretical calculated values and the experimental data are highly congruent, and the curves demonstrate excellent agreement. Specifically, when ${\text{R}}_{\text{2}}$ and${\text{R}}_{3}$ are supplied with the identical compressed gas while ${\text{R}}_{1}$ and ${\text{R}}_{4}$ remain unpressurized, or when ${\text{R}}_{\text{2}}$, ${\text{R}}_{3}$ and ${\text{R}}_{4}$ are provided with the same compressed gas while ${\text{R}}_{1}$ is not pressurized, the joint’s bending angle increases with the increase of air pressure. By comparing the two aforementioned joint compression methodologies, it can be seen that when $p_{4}=0.4\text{ MPa}$, the maximum joint bending angle is 49.5 °, representing a 12.5% increment compared to the situation when $p_{4}=0\text{MPa}$.

**Fig 19:**
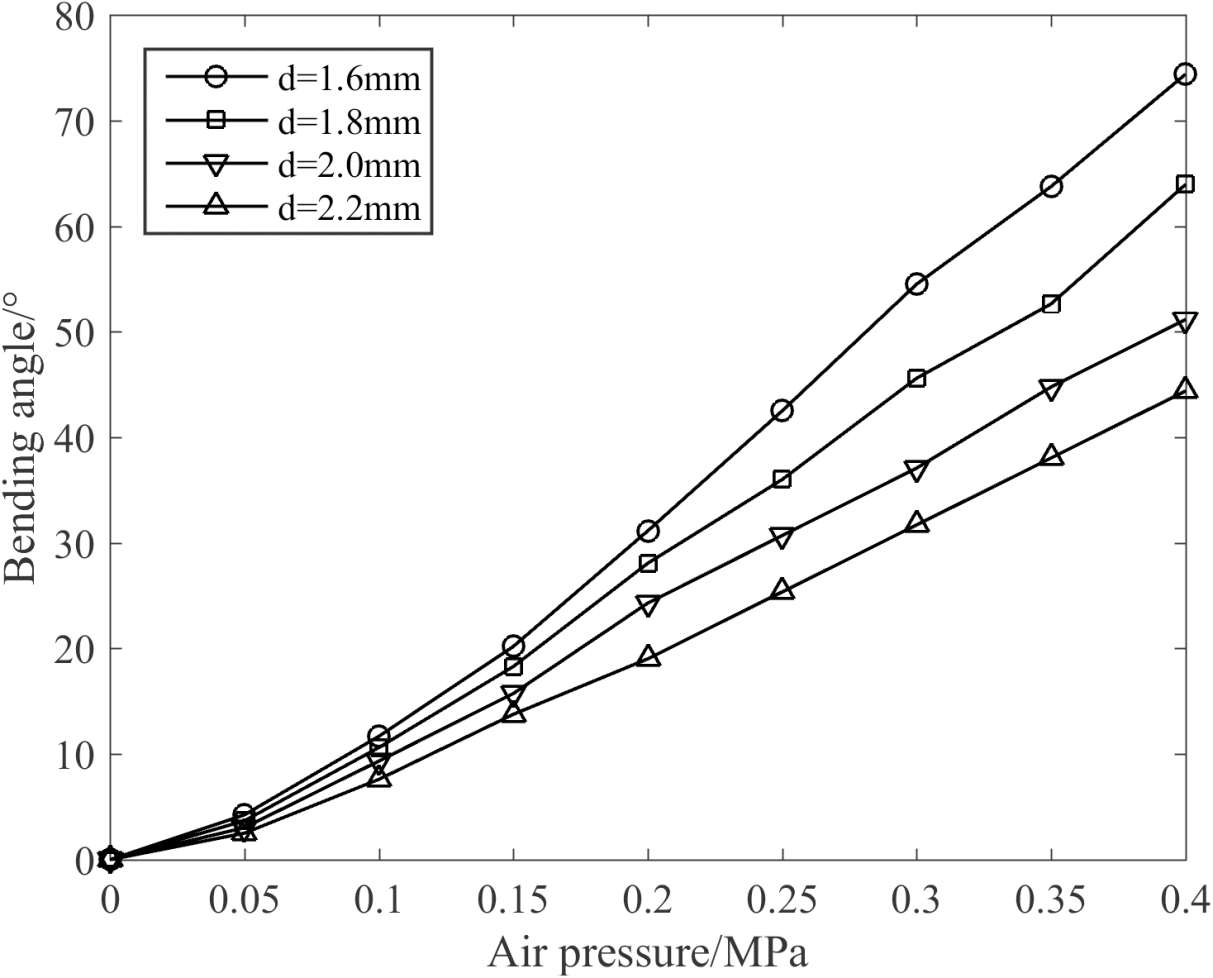
Influence of different wire diameters on the joint bending angle.

Fig 19 depicts the influence of varying wire diameters of the spring on the joint bending angle under the condition of $p_{1}=p_{4}=0\text{MPa}$,$p_{2}=p_{3}=p$. It can be clearly observed from Fig 19 that as the wire diameter grows, the flexural rigidity of the joint correspondingly rises, which in turn leads to a progressive decrease in the joint bending angle. When the air pressure is maintained at 0.4 MPa and the diameter of the wire measures 1.6 mm, and the maximum elongation of the joint reaches 74.3°.

**Fig 20:**
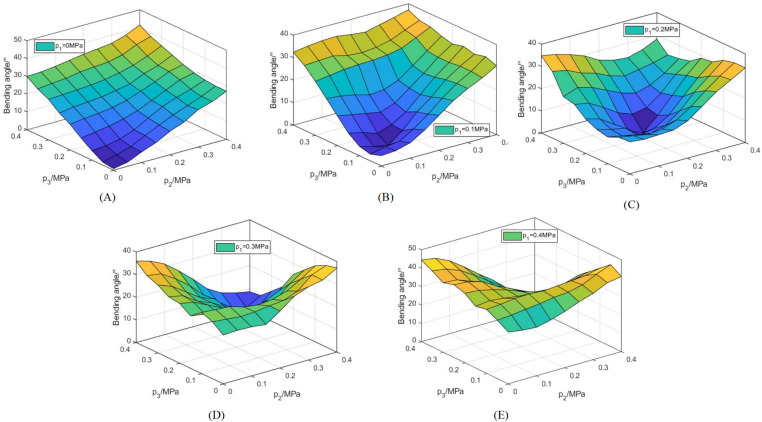
Variation curves of the joint bending angle in response to air pressure. **(A)** $p_{1}=0\text{MPa}$. **(B)** $p_{1}=0.1\text{MPa}$. **(C)** $p_{1}=0.2\text{MPa}$. **(D)** $p_{1}=0.3\text{MPa}$. **(E)** $p_{1}=0.4\text{MPa}$.

By modulating the gas pressure of artificial muscles, namely ${\text{R}}_{1}$,${\text{R}}_{\text{2}}$, and ${\text{R}}_{3}$, the joint is enabled to bend in diverse directions, and the corresponding bending angles are illustrated in Fig 20.

As depicted in Fig 20, when the air pressure of ${\text{R}}_{1}$ remains unchanged, the bending angle gradually increases and exhibits a certain nonlinearity with the increase of air pressure of ${\text{R}}_{\text{2}}$ and ${\text{R}}_{3}$. The bending angle spans a range from 0° to 44.4°. Notably, the maximum bending angle of the joint emerges in Fig 20E, that is, when $p_{1}=p_{3}=0.4\text{MPa}$ and $p_{2}=0\text{MPa}$. The minimum bending angle is 0° when $p_{1}=p_{2}=p_{3}=p$, and at this moment, the joint merely elongates without any bending.

#### 5.1.2 Experimental study on dual-muscle-driven pneumatic unidirectional bending joint.

**Fig 21:**
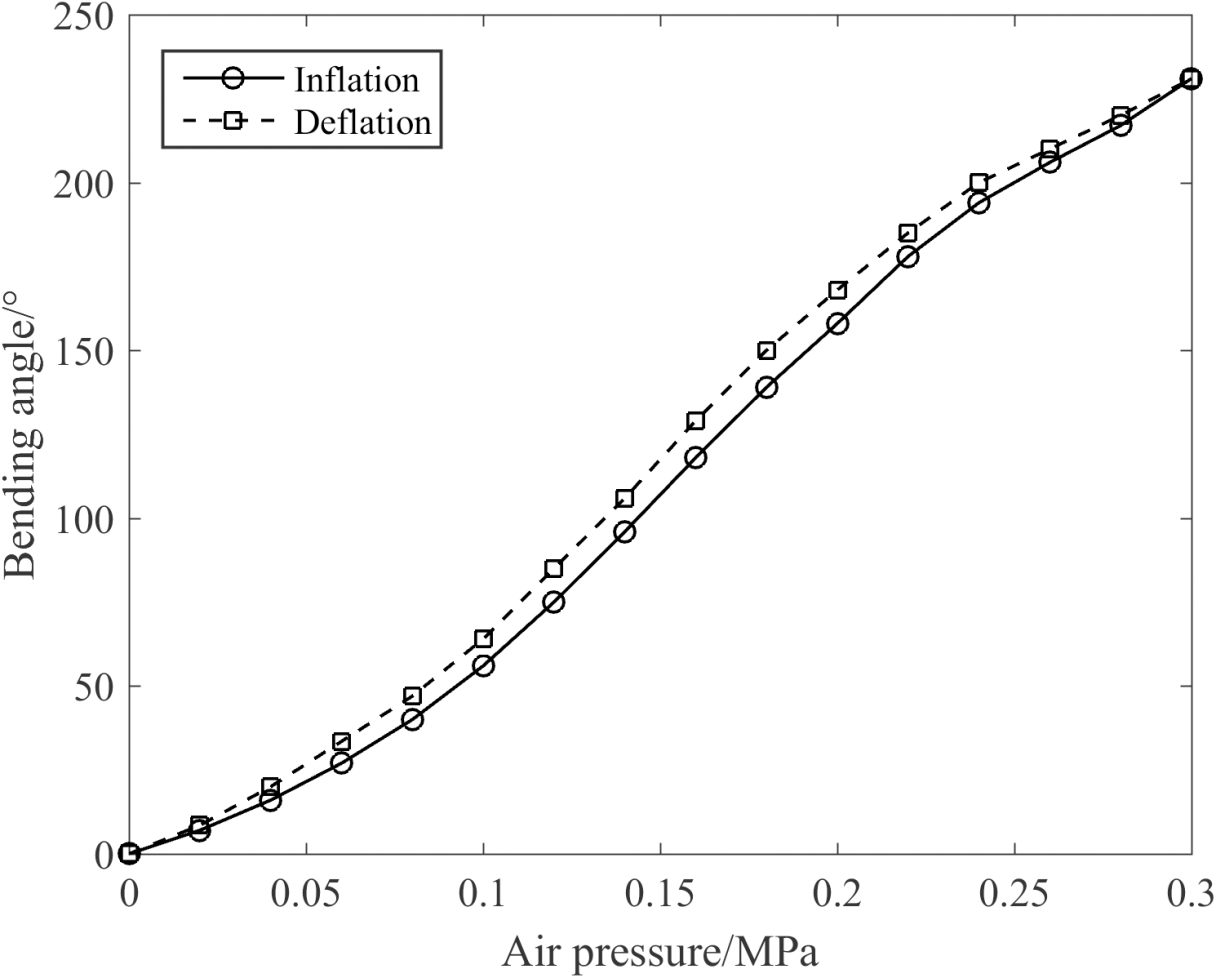
Inflation/deflation process of the joint.

The angle sensor mounted on the upper cover of the joint was employed to gauge the joint bending angles under different air pressures, as depicted in Fig 21. It is evident from Fig 21 that the joint bending angle exhibits a nonlinear increment with the rise in air pressure. Notably, the maximum bending angle reaches 231° at 0.30 MPa. Given that the silicone tube is a hyperelastic material and hysteresis occurs during the deformation process, the bending angle of the joint during the deflation stage is marginally greater than that in the inflation stage.

**Fig 22:**
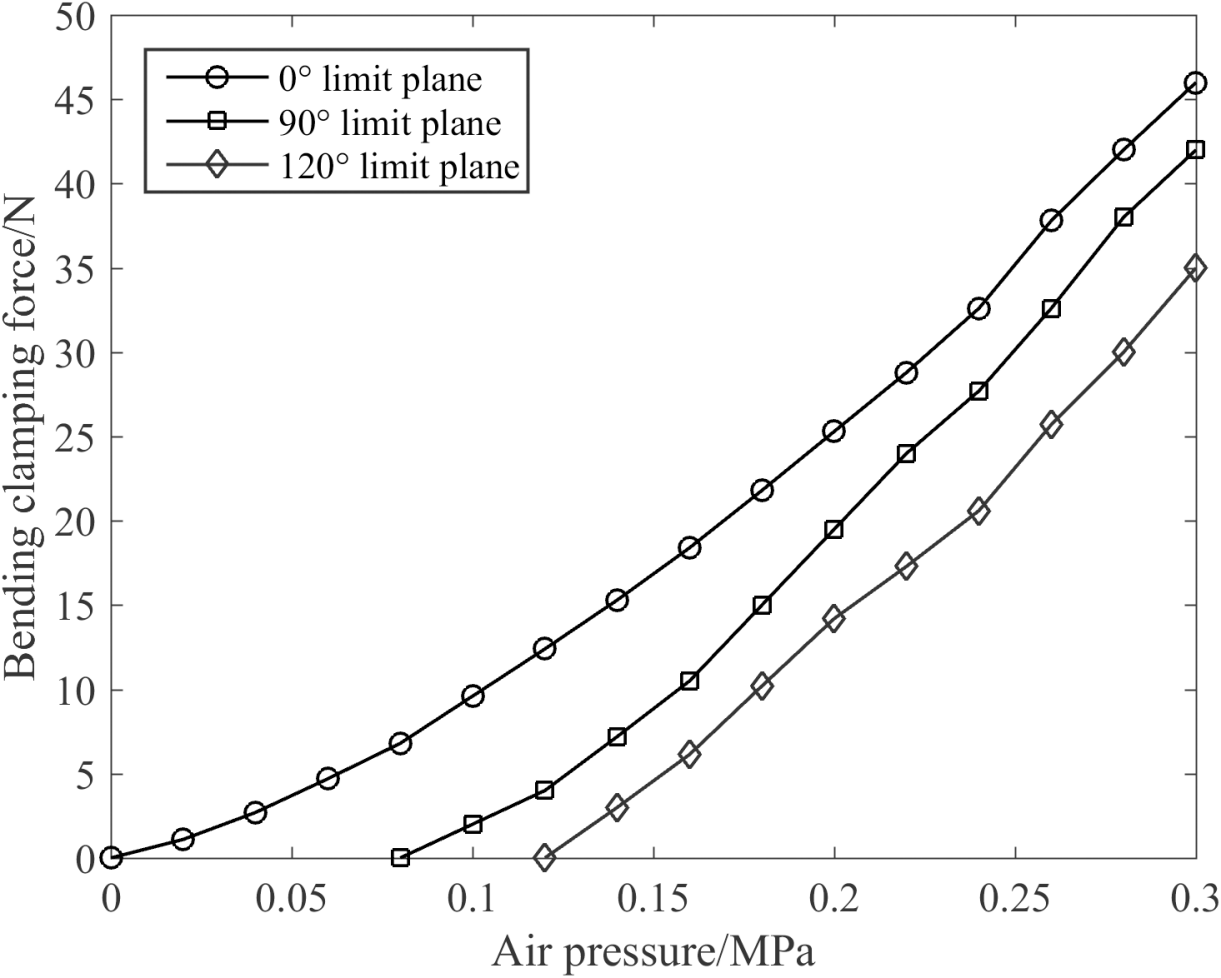
Characteristics of bending clamping force of the joint.

Once the joint undergoes bending and establishes contact with the tree, should the air pressure persist in increasing, a bending clamping force will be generated. The size of the clamping force will directly determine the stability when the robotic legs encircle the tree. More specifically, three limit planes were set in the direction of joint bending, and the bending clamping force at different bending angles was measured by employing a digital display push-pull force gauge, as depicted in Fig 22.

The following two conclusions can be derived from Fig 22. Firstly, the bending clamping force of the joint approximately increases linearly with the increase in air pressure value. At 0.30 MPa, the bending clamping force reaches 46 N. Secondly, the joint demonstrates diverse clamping capabilities corresponding to different limit planes. The fundamental principle is that the closer the limit plane is to the initial bending point, the stronger the clamping capacity of the joint becomes. Specifically, the 0° limit plane exhibits the most prominent effect. The main reason is that under an identical driving pressure, with the increase of the angle of the limiting plane, the energy consumed in the process of joint free deformation gradually increases, resulting in the weakening of its clamping ability.

### 5.2 Experiments on the driving force and anchoring force of the anchor

**Fig 23:**
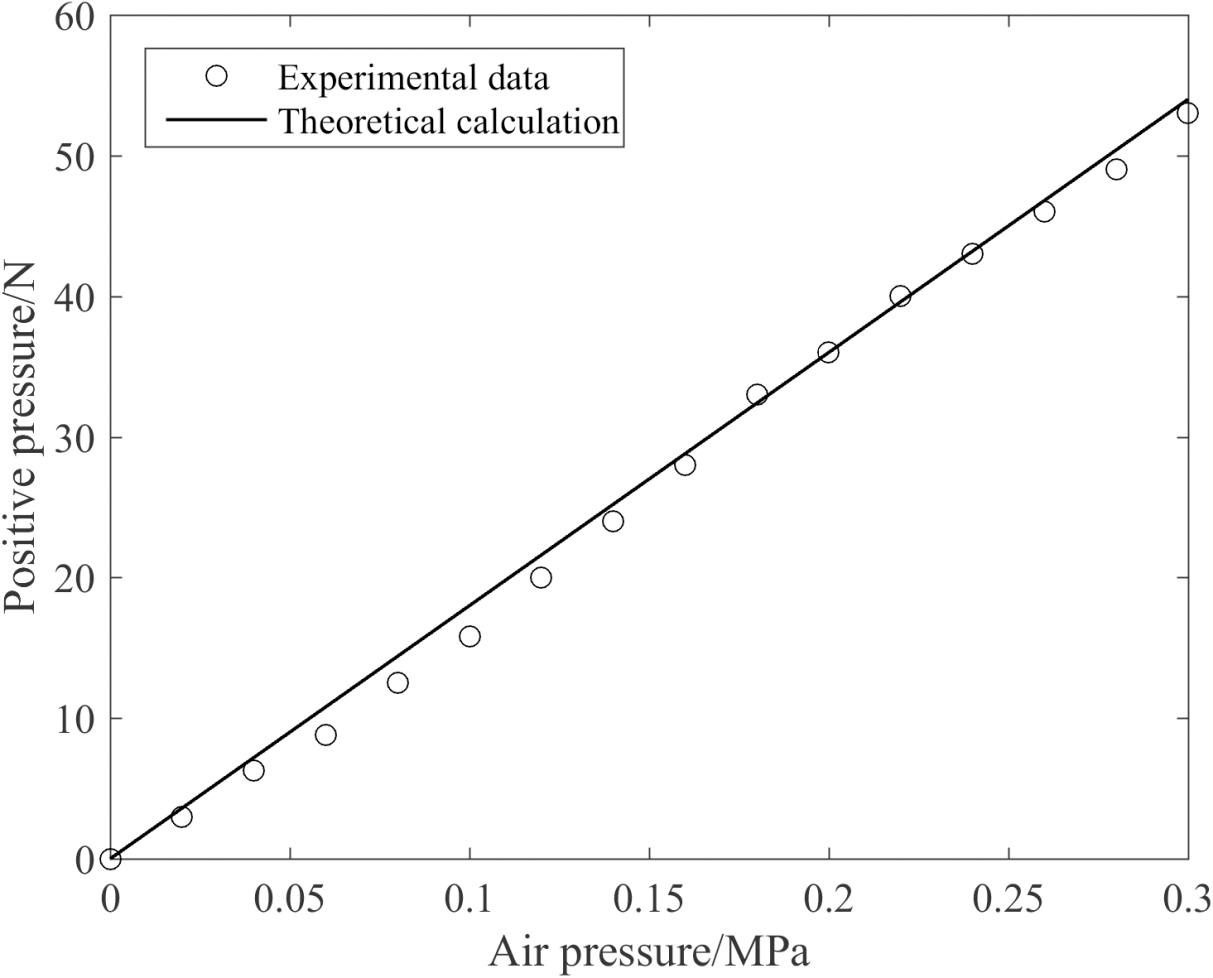
Variation of the anchor’s positive pressure with the air pressure.

Upon injecting compressed gas into the anchor, the airbag expands and deforms, propelling the moving plate and driving the needle to extend in unison. The size of the positive pressure exerted by the anchor dictates the depth to which the needle penetrates the tree surface, thus exerting a direct influence on the anchoring effectiveness. Utilize the experimental system illustrated in Fig 9 to measure the variation of the anchor’s positive pressure with respect to the air pressure precisely when the needle has just extended, as depicted in Fig 23.

It is discernible from Fig 23 that the theoretical calculation pertaining to the positive pressure exhibits a high degree of consistency with the experimental data in terms of trend, and the average error between both is 6.52%, which verifies the correctness of the simplified theoretical model. Notably, the positive pressure increases linearly with the increase in air pressure. At 0.3 MPa, the positive pressure is 53 N.

Once the needle of the anchor pierces into the tree surface, in the event that one intends to disrupt the contact between the anchor and the tree surface, thereby inducing anchoring failure, a comparatively substantial external force or load must be exerted. It is stipulated that when the sliding displacement of the anchor along the axial direction of the tree surface surpasses 1 mm or when the angle formed with the axial direction exceeds 1°, the anchor is deemed to have failed.

**Fig 24:**
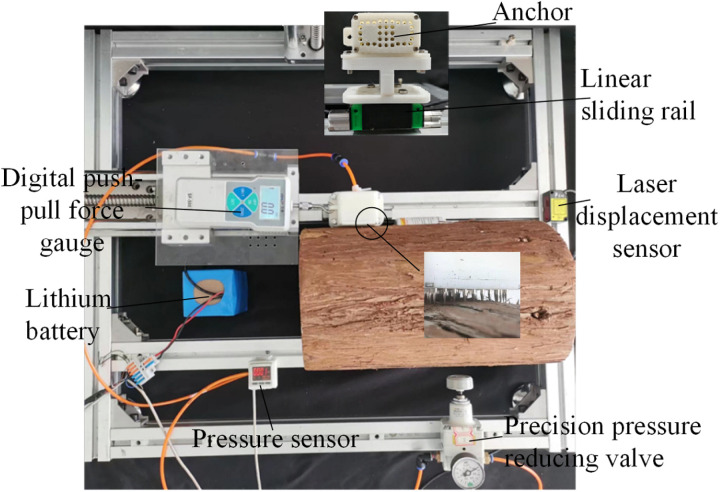
Testing device of anchoring force.

**Fig 25:**
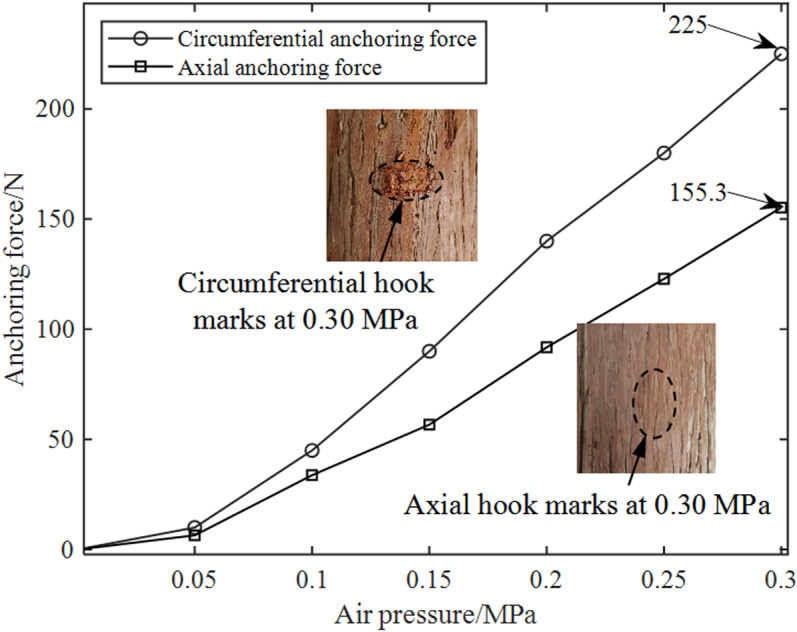
Curve of the variation of anchoring force with pressure in different directions.

In the anchoring force testing device depicted in Fig 24, the push pull force gauge is fixed on the moving slide table, with the probe hook of the force gauge being in clearance fit with the through hole on the lateral side of the anchor shell. The bottom surface of the anchor shell is connected, via a connector and bolts, to the linear slide rail that is fixed on the experimental platform. Meanwhile, the anchor plate is in direct contact with the surface of the trunk. The laser displacement sensor is installed on the experimental platform in such a way as to guarantee that the center of the sensor, the anchor center, and the axis of the force gauge probe are perfectly coaxial. Once the needle of the anchor penetrates into the tree surface, the anchor is dragged by the hook of the force gauge. Concurrently, the laser displacement sensor monitors the displacement of the anchor. The value indicated on the force gauge when the anchor fails is recorded and regarded as the maximum anchoring force that the anchor is capable of providing under the current air pressure. By altering the placement orientation of the trunk, the data concerning the variation of the anchoring force in different directions in relation to the air pressure was measured, as illustrated in Fig 25.

The following two conclusions can be drawn from Fig 25. Firstly, the anchoring force increases with the increase of air pressure. The main reason is that with the increase of air pressure, the positive pressure of the anchor correspondingly increases. This, in turn, leads to a deeper penetration of the needle into the tree, ultimately resulting in a greater pulling force or load required to induce anchoring failure. Secondly, due to the influence of the tree surface texture, under the same air pressure conditions, the circumferential anchoring force of the anchor surpasses the axial anchoring force. At 0.30 MPa, the maximum circumferential anchoring force can reach 225 N, whereas the maximum axial anchoring force is 155.3 N.

### 5.3 Experiment study on the clamping performance of the leg

#### 5.3.1 The range of tree diameters adaptable by the leg.

**Fig 26:**
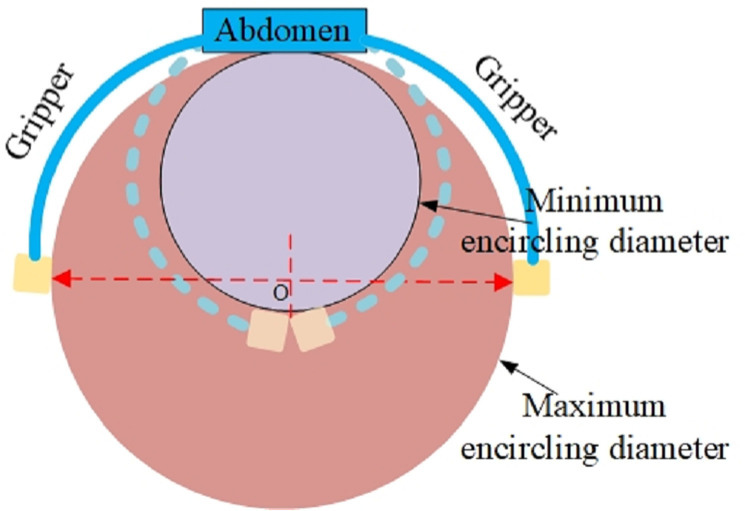
Analysis of the adaptable range of tree diameters.

In order to guarantee the stability of the robot during its movement, the leg ought to encircle at least half of the tree’s cross-section circumference. This way, a more favorable force-closure effect can be achieved, and such a diameter represents the maximum tree diameter that the leg can encircle. When the leg undergoes bending and deformation to completely encircle the tree for one full turn, a shape closure is formed, which corresponds to the minimum tree diameter that the leg can encircle. If the tree diameter diminishes further, interference will occur between the leg end anchors, as illustrated in Fig 26.

It can be deduced from the analysis of Fig 26 that the range of variation in tree diameter to which the leg can adapt is presented as follows:


$$\begin{array}{c} \left\{ \begin{array}{c} \frac{\;\;\;\pi \;\;\;{\text{D}}_{\text{max}}}{\text{2}}=\text{2}{\text{L}}_{\text{g}}+\text{0.5}{\text{L}}_{\text{f}}\\ \;\;\;\pi \;\;\;{\text{D}}_{\text{min}}=\text{2}{\text{L}}_{\text{g}}+\text{0.5}{\text{L}}_{\text{f}} \end{array}\right. \end{array}$$ (28)

Where ${\text{D}}_{\text{max}}$ is the maximum encircling tree diameter, ${\text{D}}_{\text{min}}$ is the minimum encircling tree diameter, ${\text{L}}_{\text{g}}$ is the length of the gripper, ${\text{L}}_{\text{f}}$ is length of the abdominal anchor.

Substituting the dimensional parameters of the leg joint and anchors into Equation (28), it can be determined that the range of tree diameter variation to which the leg can adapt is 146.4 mm to 292.8 mm.

#### 5.3.2 Experiment on clamping characteristics of the leg.

**Fig 27:**
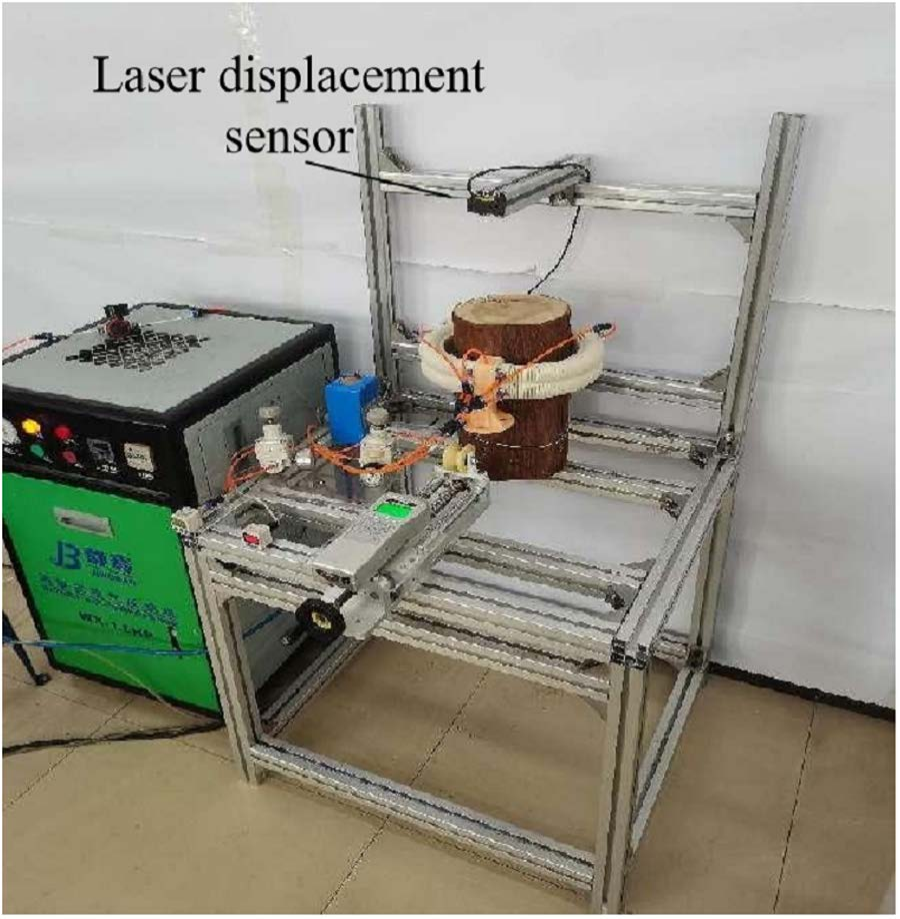
Test device for axial load on leg.

In the process of encircling and clamping, the leg predominantly depends on the bending clamping force generated during the joint’s encircling motion and the anchoring force produced by the anchor to counterbalance its own weight and external loads. Fig 27 illustrates the experimental setup designed for measuring the leg’s axial load. A trunk with a diameter of 190 mm is vertically affixed to the experimental platform, while the force gauge is installed on the moving sliding platform. The two ends of the rope are respectively connected to the leg connectors and the center of the force gauge. By means of the guiding function of pulleys, it is ensured that the direction in which the leg endures the load remains perpetually vertical.

**Fig 28:**
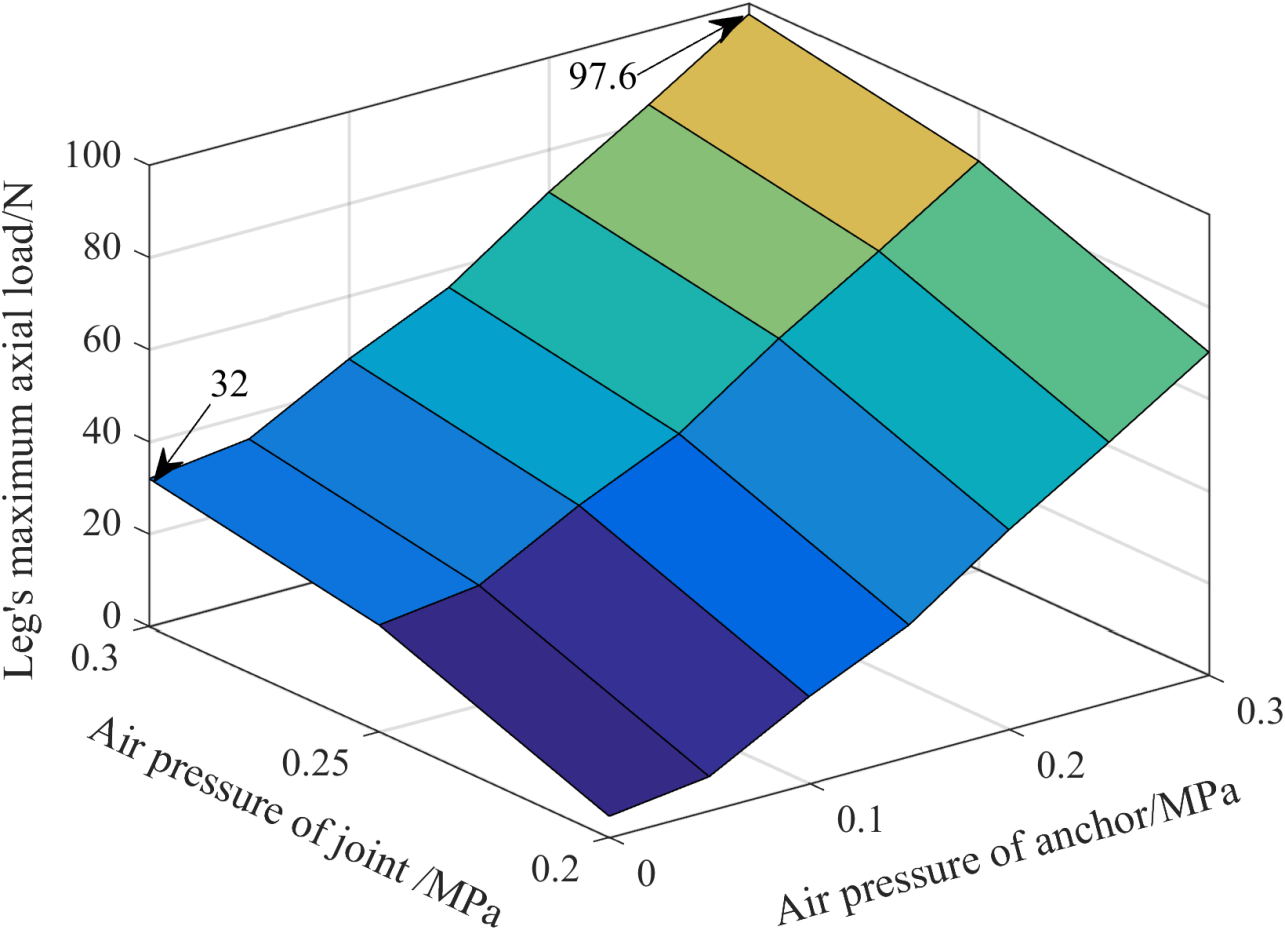
Curved surface of axial load on legs with changes in air pressure.

During the experiment, the continuous variation of the leg load is achieved by towing the force gauge with the moving sliding platform. Simultaneously, the laser displacement sensor is employed to monitor the axial displacement fluctuations between the leg and the tree. When the displacement reaches the specified value for anchoring failure, the data of the force gauge is immediately recorded and regarded as the maximum load that the leg can bear under this air pressure combination, as depicted in Fig 28.

As can be discerned from Fig 28, the maximum axial load that the leg can bear gradually increases with the increase of the air pressure of the joint and the anchor, and the load varies from 4.57 N to 97.6 N. When the anchor remains inactive, the maximum axial load of the leg is 32 N. However, when the anchor and joint air pressure are both 0.3 MPa, the maximum load that the leg can endure amounts to 97.6 N. Compared with the situation where the anchor is non-operational, the maximum load has witnessed a remarkable increase by 3.05 times.

### 5.4 Motion performance experiments of the tree-climbing robot

The experimental system depicted in Fig 10 was employed to carry out the robot kinematics experiment. Through programming the Programmable Logic Controller to modulate the size of the electromagnetic proportional valve as well as the on-off status of the electromagnetic reversing valve, the robot can achieve the climbing action on the tree in accordance with the gait planning. During the experimentation, the 3D motion capture system is utilized to measure and obtain the changes in the robot’s movement displacement under different air pressure, tree diameter, tree inclination angles and loads. The specific experimental conditions are presented in Table 4.

**Table 4:** Experimental conditions.

Parameter	Numerical value	Unit
Working air pressure of waists	0.2, 0.3, 0.4	MPa
Sampling time	200	s
Inclination angle of climbing tree	0, 30, 60, 90	°
Diameter of climbing tree	150, 220, 290	mm
Load	0, 1.0, 2.0	kg

#### 5.4.1 Experiment of robot variable diameter adaptability.

**Fig 29:**
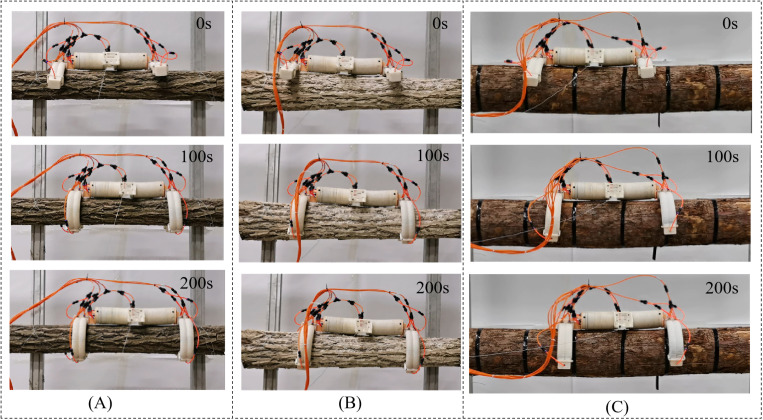
Climbing attitudes of various tree species and diameters. (A) 150mm diameter willow tree. (B) 220mm diameter elm tree. (C) 290mm diameter pine tree.

In order to validate the climbing performance of the robot on trees that possess diverse diameters within the theoretical calculation scope, a selection of various tree species with differing diameters was made for climbing experiments, as illustrated in Fig 29.

**Fig 30:**
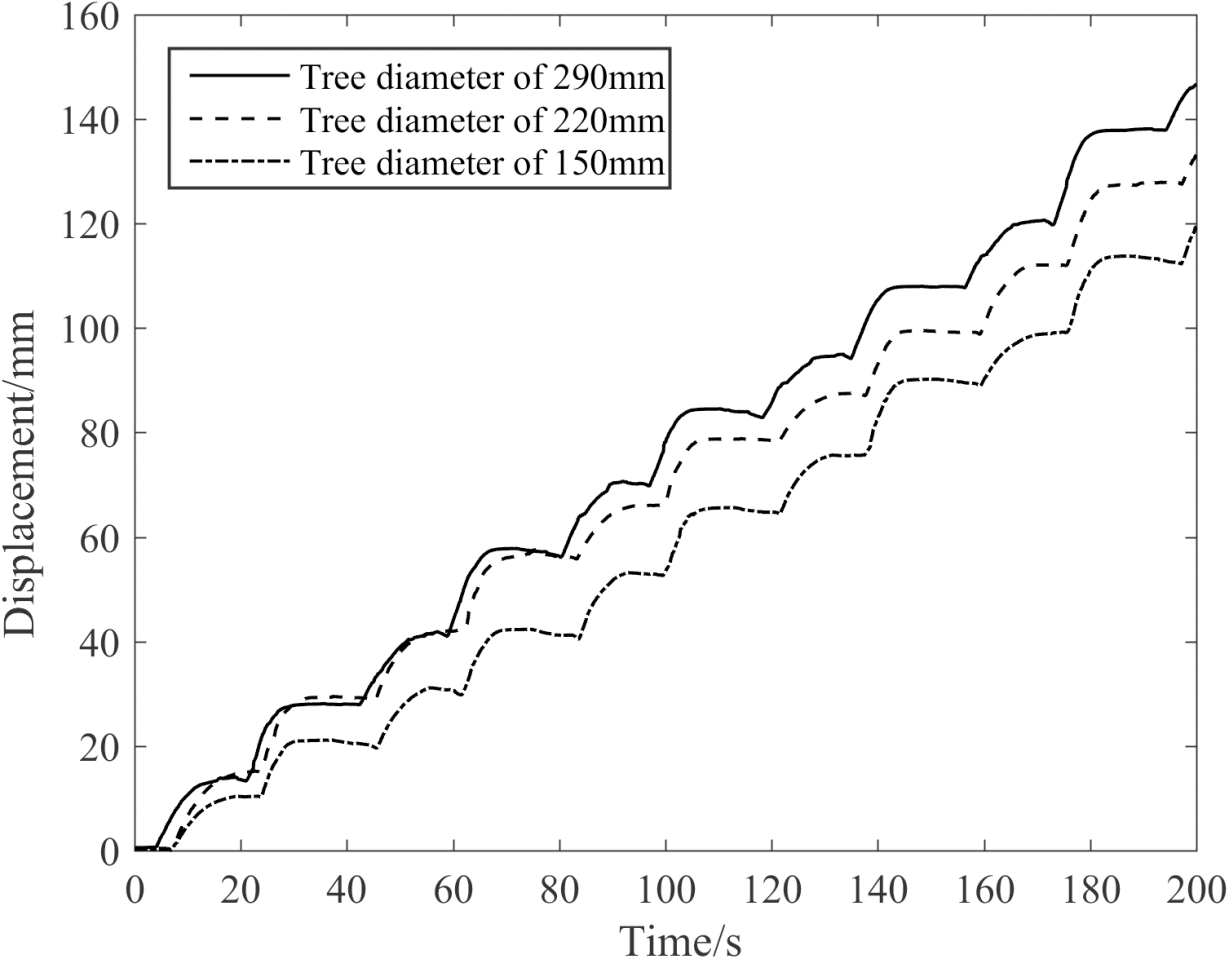
Influence of varying tree diameters on the robot’s horizontal crawling displacement.

Fig 30 illustrates the correlation between the horizontal climbing displacement of the robot under no-load conditions and time, with the waist joint operating at 0.4 MPa on trees of varying diameters. It is evident from Fig 30 that the robot demonstrates the capability to adapt to climbing on trees with diameters spanning from 150 mm to 290 mm. Within this adaptable diameter range, as the tree diameter enlarges, the movement speed of the robot increases correspondingly. When climbing a tree with a diameter of 290 mm, the crawling speed reaches 43.8 mm/min. The underlying reason is that as the tree diameter grows, the time required for the robot’s legs to complete the encircling deformation diminishes, shortening the cycle for the robot to complete a horizontal crawling gait and consequently accelerating its speed.

#### 5.4.2 Robot obstacle-crossing experiment.

**Fig 31:**
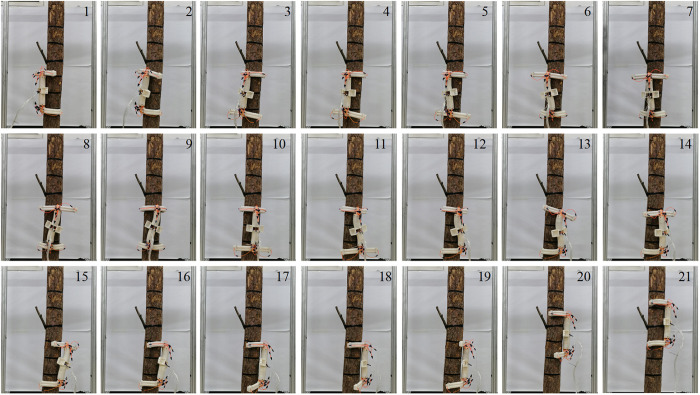
The process of robot crossing obstacle.

During the tree-climbing process, when the robot comes across obstacles like branches or burls, it must adhere to the preset obstacle-crossing gait to circumnavigate the impediments and carry on with its crawling. In light of the size and shape of obstacles, the robot is required to modulate the rotation angle within the obstacle-crossing gait. Fig 31 depicts the process of the robot crossing obstacles under the given size of obstacles and rotation angle. Fig 31 (1–16) represent three complete cycles of tree -climbing and obstacle-crossing, with a rotation angle of 40° in each rotation cycle. Fig 31 (17–21) illustrate the process of the robot climbing up the tree by means of the upward climbing gait subsequent to evading the obstacle.

#### 5.4.3 Kinematic experiments of the robot under different working conditions.

(1) Influence of varying tree inclination angles on robot’s upward tree-climbing

**Fig 32:**
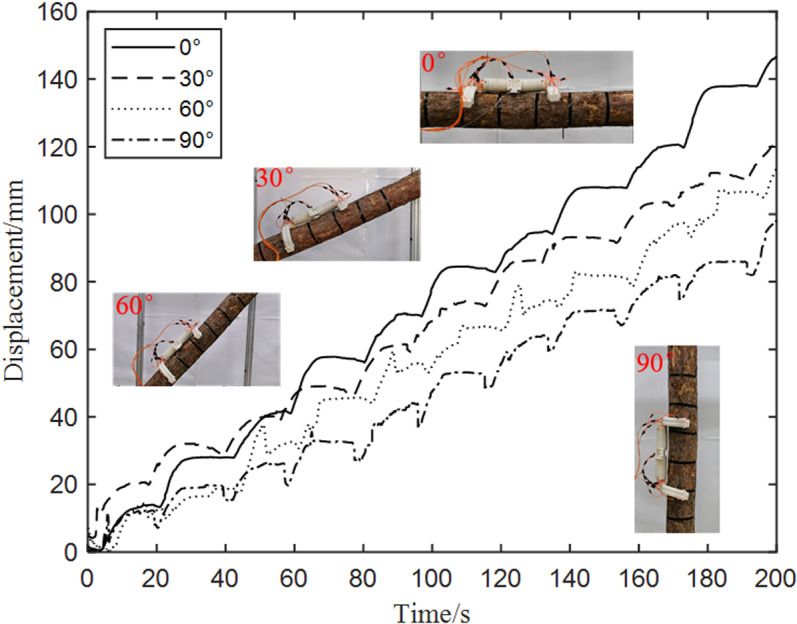
Influence of varying tree inclination angles on robot’s upward tree-climbing.

Fig 32 illustrates the influence of different inclination angles on the upward displacement of the robot when its waist is actuated with an air pressure of 0.4 MPa while climbing pine trees 290 mm in diameter under unloaded conditions.

It can be seen from Fig 32 that as the inclination angle of the tree increases, the climbing speed of the robot gradually decreases. When the inclination angles of the tree are 0°, 30°, 60° and 90° in turn, the corresponding upward speeds of the robot are 43.8 mm/min, 36 mm/min, 32.4 mm/min, and 27 mm/min respectively. The underlying cause is that as the inclination angle of the tree increases, the gravitational component that the robot must contend with grows larger. Simultaneously, the elongation and rebound magnitudes of the robot’s waist joint shrink, thereby precipitating a slower movement speed for the robot.

(2) Influence of air pressure on robot’s upward tree-climbing

**Fig 33:**
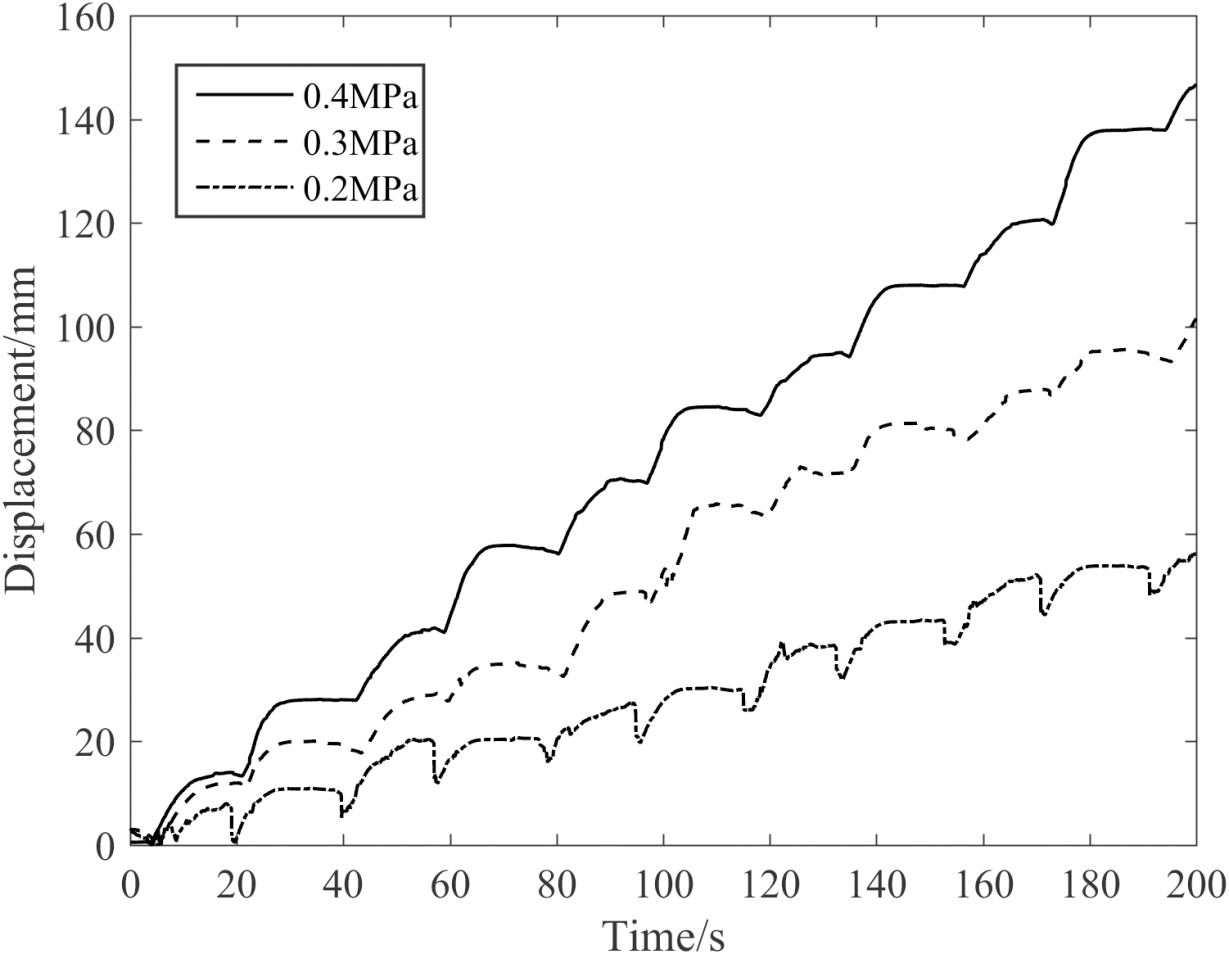
Influence of air pressure on the robot’s horizontal crawling displacement.

Fig 33 presents the influence of varying waist air pressures on the upward speed of the robot when climbing a pine tree with a diameter of 290 mm and a tree inclination angle of 0 ° in an unloaded state. Evidently from Fig 33, as the robot’s walking step length expands with the rise in waist air pressure, its climbing speed concomitantly increases. In comparison to the climbing speed at 0.2 MPa, the climbing speeds at 0.3 MPa and 0.4 MPa are augmented by 1.45 times and 2.64 times respectively.

(3) Influence of load on robot’s tree-climbing displacement

**Fig 34:**
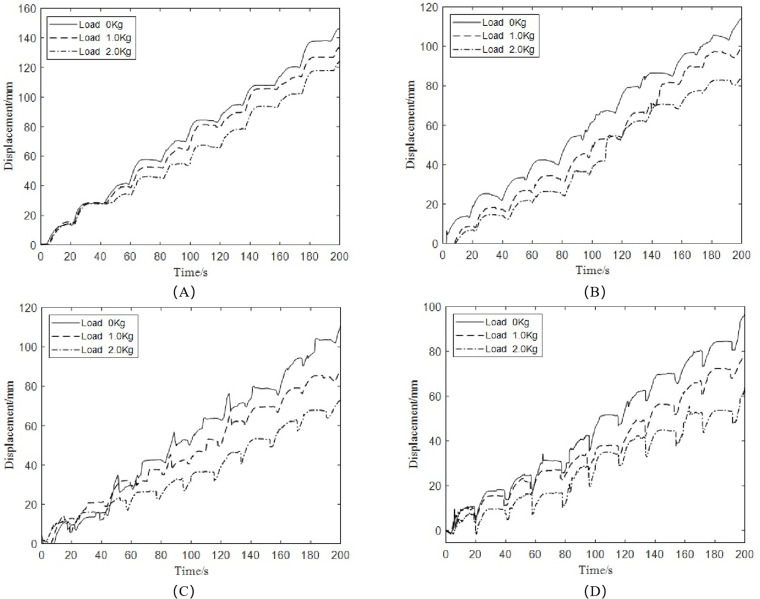
Influence of load on robot’s tree-climbing displacement (A) 0°. (B) 30°. (C) 60°. (D) 90°.

Fig 34 depicts the influence of different loads on the robot’s crawling displacement when it is driven at the waist with an air pressure of 0.4 MPa while climbing pine trees with a diameter of 290 mm under unloaded conditions. It can be seen from Fig 34 that under the same tree inclination angle, the robot’s walking step distance gradually decreases with the increase of load, leading to a decline in its climbing speed.

**Fig 35:**
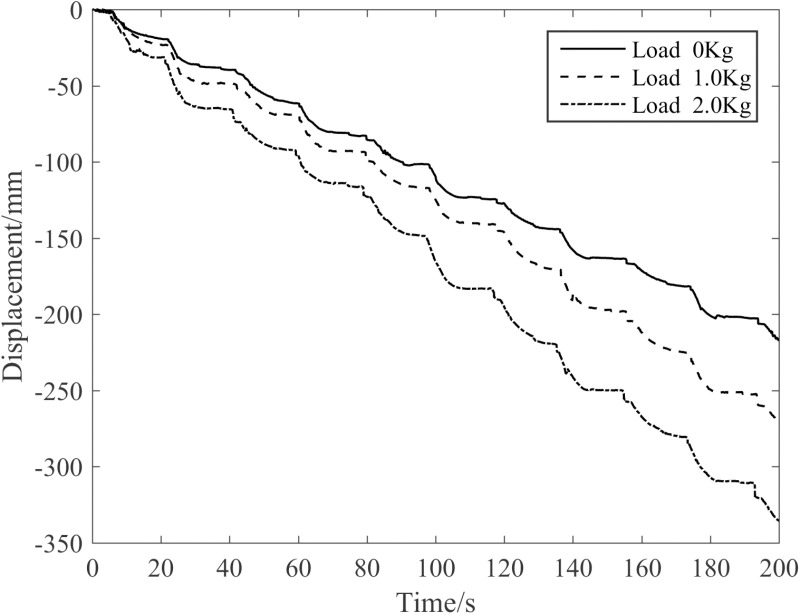
Influence of load on robot’s downward tree-climbing displacement.

Fig 35 presents the influence of different loads on the robot’s downward displacement when the robot, with its waist driven at 0.4 MPa, climbs 290-mm-diameter pine trees. It’s apparent from Fig 35 that as the load increases, the elongation of the waist becomes greater, which in turn expands the downward step length during tree-climbing. Consequently, the robot’s downward climbing speed rises correspondingly with the increasing load. In comparison to the speed under no-load conditions, the crawling speeds of the robot under loads of 1.0 kg and 2.0 kg are enhanced by 1.29 times and 1.55 times respectively.

**Fig 36:**
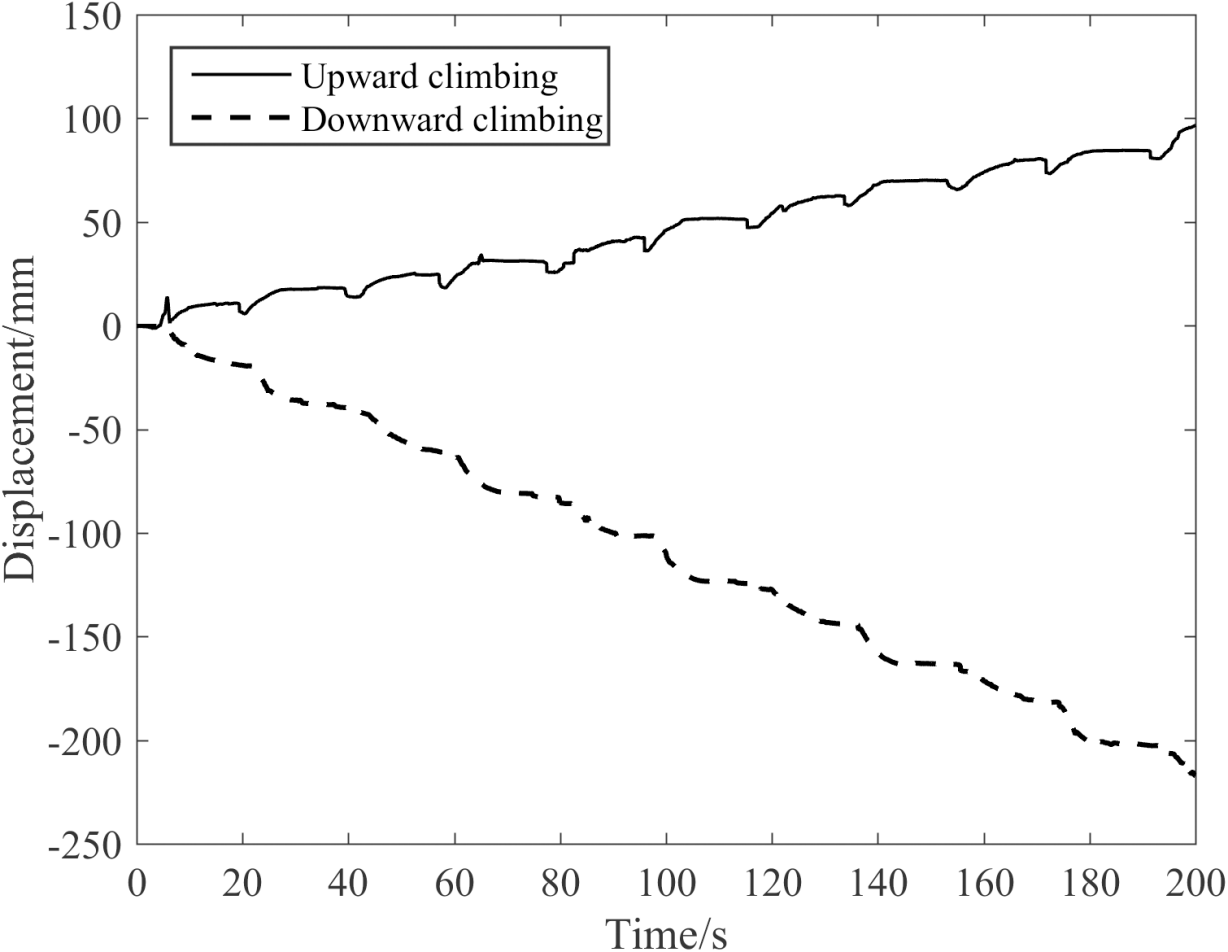
Comparison of upward and downward displacements during vertical tree-climbing.

Fig 36 illustrates the comparison of the upward and downward displacements of the robot when climbing a pine tree with a diameter of 290 mm in an unloaded state and with the waist-driven air pressure at 0.4 MPa.

As can be seen from Fig 36, when the robot climbs trees under no-load conditions, its downward speed is 63 mm/min, while its upward speed is 27 mm/min. The main reason for the downward speed of climbing trees being greater than the upward speed is that the robot’s self- weight plays a completely opposite role due to the distinct crawling directions. When climbing trees downward, the direction of the robot’s gravity aligns with that of the waist elongation, augmenting the elongation. Conversely, when climbing trees upward, the robot’s own weight acts as a load, and the direction of its gravity opposes that of the waist elongation, impeding the elongation process.

## 6 Conclusion

In this paper, a novel pneumatic flexible tree-climbing robot is devised by leveraging the self-developed pneumatic flexible joints and retractable needle anchors. Through the air pressure control system, by coordinating waist deformation and leg’s encircling and clamping alternately, the robot can carry loads to complete the functions of climbing up trees, climbing down trees and crossing obstacles at different incline angles. The structure and working principle of the robot are elaborated in detail, the joint theoretical model is established, and a series of experiments are conducted. The conclusions drawn are as follows:

(1) An enhanced pneumatic flexible spatial bending joint with 3 degrees of freedom was designed. The mechanical models of the joint’s deformation and driving were established and experimentally verified. The experimental results show that both the elongation and the bending angle of the joint increase nonlinearly with the increase of the air pressure value, and the trends of the theoretical calculations and the experimental data are consistent and in good agreement. By adjusting the internal cavity air pressure of artificial muscles ${\text{R}}_{1}$, ${\text{R}}_{2}$ and ${\text{R}}_{3}$ within the joint, it can generate axial elongation and spatial bending deformation in any direction from 0° to 360°. Once the enhanced artificial muscle ${\text{R}}_{4}$ is injected with compressed gas, it has an amplifying effect on the deformation magnitude and driving force of the joint. In comparison to the unenhanced state, when $p_{4}=0.4\text{ MPa}$, the joint elongation is 35.4 mm, with an increase of 14.2% in the elongation, the axial driving force reaches 296 N, reflecting a 6.5% increase, and the bending angle is 49.5°, indicating a 12.5% increment.
(2) A gripper with controllable bending angle and anchoring force was devised, and corresponding experimental studies were carried out. The experimental results show that the bending angle of the gripper is 231°and its maximum bending force is 46 N at 0.3 MPa. Moreover, the positive pressure and anchoring force of the anchor increase continuously as the air pressure rises. At 0.3 MPa, the maximum positive pressure is 53 N, the circumferential anchoring force is 255 N, and the axial anchoring force is 155.3N.
(3) Three gaits enabling the robot to climb up trees, climb down trees and cross obstacles were planned, and the rationality of gait planning was experimentally validated. The prototype of the robot’s leg was designed and fabricated. Subsequently, the diameter range of the tree that the leg can encircle was analyzed, and a clamping performance experiment of the leg was carried out. The experimental results show that the robot’s leg can adapt to the stable gripping of various types of trees within a diameter range of 146.4 mm to 292.8 mm. Within this interval, the robot’s climbing speed increases gradually with the increase of the diameter. When encircling a pine tree with a diameter of 190 mm, the maximum axial load that the leg can bear varies from 4.57 N to 97.6 N.
(4) The kinematics experiments of the robot were conducted, and its motion performance under different air pressures, tree diameters, tree inclination angles and load conditions were obtained. The experimental results indicate that the robot can adapt to climbing trees with inclination angles ranging from 0° to 90°, with a maximum load mass of 2 kg. As the tree inclination angle and load increase, the robot’s climbing up speed gradually decreases, but the load exerts a positive facilitating effect on the downward climbing speed. When encircling a pine tree with a diameter of 290 mm, the maximum horizontal crawling speed is 43.8 mm/min under no-load conditions, the maximum climbing up speed is 27 mm/min at a 90° vertical angle, and the climbing down speed is 63 mm/min.

Certainly, the robot developed in this paper still has deficiencies in terms of its own weight, climbing speed, and control, and requires further optimization work. Future work may include, but is not limited to, the following aspects:

(1) Further optimize the waist and leg joints to reduce the robot’s own weight, achieve lightweight design, and enhance the driving ability of the joints and the climbing speed.
(2) Further establish a kinematic theoretical model of the entire robot to provide support for more flexible and reliable climbing movements.
(3) Integrate flexible sensors onto the robot to endow it with proprioceptive and exteroceptive capabilities, realize closed-loop or semi-closed-loop autonomous adjustment and control, and create a more autonomous and adaptable robot.

The optimized tree-climbing robot is expected to have broad application prospects in forest inspection, survey, information collection, etc.
